# Anatomy of the Female Koala Reproductive Tract

**DOI:** 10.3390/biology12111445

**Published:** 2023-11-17

**Authors:** Sara Pagliarani, Chiara Palmieri, Michael McGowan, Frank Carrick, Jackson Boyd, Stephen D. Johnston

**Affiliations:** 1 Ontario Veterinary College, The University of Guelph, Guelph, ON N1G 2W1, Canada; 2 School of Veterinary Science, The University of Queensland, Gatton 4343, Australia; 3 School of Environment, The University of Queensland, Gatton 4343, Australia; 4 Sustainable Minerals Institute, Koala Study Program, The University of Queensland, St. Lucia 4072, Australia

**Keywords:** koala, ovary, reproductive tract, anatomy, estrous cycle

## Abstract

**Simple Summary:**

The koala has recently been listed as an endangered species in the northern part of its range where it faces significant challenges associated with habitat fragmentation, climate change, and disease. Fundamental to its conservation is a detailed knowledge of its reproductive system, particularly with respect to what represents normal anatomy and/or pathology. In order to conserve we must first understand. This article brings together findings from two PhD theses that provide a detailed description of the female koala reproductive anatomy and histology through different presumptive stages of the reproductive cycle.

**Abstract:**

The koala (*Phascolarctos cinereus*), while being an iconic Australian marsupial, has recently been listed as endangered. To establish an improved understanding of normal reproductive anatomy, this paper brings together unpublished research which has approached the topic from two perspectives: (1) the establishment of an artificial insemination program, and (2) the definition of *Chlamydia* spp.-derived histopathological changes of the female koala urogenital system. Based on the presentation and histological processing of over 70 opportunistic specimens, recovered from wildlife hospitals in Southeast Queensland (Australia), we describe the gross and microanatomy of the koala ovary, oviduct, uteri, vaginal complex, and urogenital sinus during the interestrous, proliferative, and luteal phases of the reproductive cycle.

## 1. Introduction

The female reproductive tract of the koala is similar to other marsupials with the presence of a urogenital sinus opening into a cloaca, two lateral vaginae, a partitioned vaginal cul-de-sac, and duplex cervices and uteri. The two lateral vaginae with the vaginal cul-de-sac may together be referred to as the vaginal complex [1,2,3]. Studies of the reproductive anatomy of the female koala have been undertaken as long ago as Forbes (1881) [1], O’Donoghue (1916) [2], and Mackenzie (1919) [3], and reviewed by Handasyde (1986) [4] and Brown (1987) [5]; subsequent reviews [6,7,8,9] have added few details to these former descriptions. The majority of work has mostly concerned the gross morphology of the reproductive tract, with the exception of Brown [5], Obendorf [10], and Handasyde et al. [11], who also reported histological observations. However, Obendorf [10] and Handasyde et al. [11] described histological changes in the reproductive tract of female koalas after injection of estradiol, and only the PhD research conducted by Johnston [12] and Pagliarani [13] has attempted to provide detailed descriptions of gross and histological changes of the ovary and the reproductive tract during the reproductive cycle. 

Given the koala’s current endangered status listing in Queensland, New South Wales, and the Australian Capital Territory [14], and the high incidence of *Chlamydia* spp. in this species, obtaining well-preserved reproductive tissues from unaffected koalas has been challenging. Nevertheless, before it is possible to interpret the effects of gross and histological changes to the reproductive tract caused by diseases such as chlamydiosis, the normal anatomy of each region of the reproductive tract (oviduct, uterus, cervix, vaginal cul-de sac, lateral vaginae, and urogenital sinus) at different stages of the reproductive cycle must be well defined. 

The present article collates the findings from two separate doctoral theses, one focused on the development of assisted breeding technology in the koala [12], and a more recent study which described the pathology of chlamydial disease in the reproductive tract of female koalas [13]. The primary objective of this article was to integrate the collective knowledge of female koala reproductive anatomy obtained from both studies to better define the gross and microanatomical structure of the reproductive tracts of wild koalas broadly at different stages of the reproductive cycle. The determination of the different stages of the reproductive tract was entirely based on the gross and histological assessment of ovarian and uterine physiological status, as the measurement of systemic steroid hormone secretion [15] was not possible, nor will this mini review focus on describing koala ovarian follicular or luteal dynamics.

## 2. Materials and Methods

### 2.1. Animals

As part of Johnston’s PhD research [12], a total of 50 female adult koala bodies were obtained from the Queensland National Park and Wildlife Service’s (QNPWS) Koala Hospital, (Moggill, Southeast Queensland) between June 1996 and March 1997, and examined for gross anatomy. Following an initial post-mortem examination by QNPWS staff, koala bodies were frozen and stored at −20 °C for approximately 1 week. Female koalas showing gross evidence of urogenital tract pathology, including bursitis, salpingitis, metritis, vaginitis, or cystitis, were not examined. The pouch was checked for the presence of young and/or evidence of recent lactation, as indicated by an enlarged mammary gland or elongated teats. A further 6 koala reproductive tracts were obtained from Lone Pine Koala Sanctuary (LPKS) (Fig Tree Pocket, Southeast Queensland) from January to June 1997, and examined for histology by fixing their reproductive tracts in 10% buffered formalin within 2 h of euthanasia. As part of Pagliarani’s PhD thesis [13], a further 20 adult female koala cadavers were obtained from Currumbin Wildlife Hospital (Currumbin, QLD, Australia) and Australia Zoo Wildlife Hospital (Beerwah, QLD, Australia) between July 2017 and May 2020; the reproductive tracts evaluated in Pagliarani’s thesis were obtained from those koalas which were recently dead on arrival at the hospital or were subsequently euthanized due to welfare issues. None of the 20 koalas showed any gross evidence of disease or pathology in their reproductive tract. The pouch was checked for the presence of young and/or evidence of recent lactation, as indicated by an enlarged mammary gland or elongated teats. Both studies [12,13] were conducted under the approval of The University of Queensland Animal Ethics Committee (Approval number: ANRFA/SVS/335/17).

### 2.2. Gross Reproductive Anatomy during Different Stages of the Reproductive Cycle

**Figure 1:**
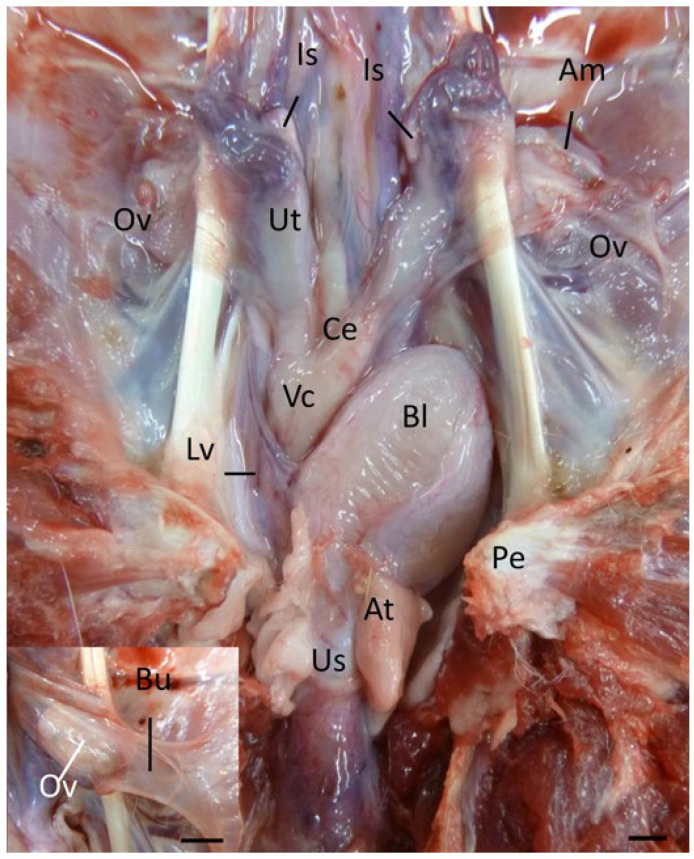
In situ location of the female koala reproductive system. Insert—Ovary within the ovarian bursa. Am—Ampulla of oviduct, At—Adipose tissue in pelvic canal, Bl—Bladder, Bu—Ovarian bursa, Ce—Cervix, Is—Isthmus of oviduct, Lv—Lateral vagina, Pe—Pelvic floor bisected, Ov—Ovary, Vc—Vaginal cul-de-sac, Us—Urogenital sinus. Bar = 1 cm. Inset: Bar = 0.5 cm.

Following the removal of the ventral portion of the pelvic floor to access the urogenital sinus and lower vaginal complex (Figure 1), the complete reproductive tract (including the ovarian bursa) was removed, pinned carefully into position, and immersed and fixed in 10% buffered (pH 7.2) formalin. After fixation for a period of 3 d, each tract was cut into 5 mm transverse sections from the caudal extremity of the urogenital sinus to the point at which the lateral vaginae entered the upper vaginal cul-de-sac. The remainder of the vaginal cul-de-sac/uterine complex was then cut longitudinally in a horizontal plane to expose the internal anatomy of the uteri and their structural relationship to the upper vaginal cul-de-sac. The urogenital sinus, lateral vaginae, and vaginal cul-de-sac in each 5 mm transverse section of the reproductive tract were examined under a stereo microscope (×10), and cross-sectional dimensions measured using vernier calipers (±0.05 mm). The precise measurement of the oviduct was difficult given the convoluted anatomy of the isthmus and the difficulty of observing the ampullary region of the oviduct, which was concealed by the fimbriated folds of the infundibulum. Direct measurements of what MacKenzie [3] and Brown [5] described as the straight (ampulla, *n* = 22) and convoluted (isthmus and extramural junctura, *n* = 26) components of the oviduct were recorded. The lineal measurements of each component of the female koala reproductive tract have been defined by Johnston [12]. For the purposes of this study, each uterus was considered to consist of 2 functional units: a glandular portion characterized by the presence of endometrium, and an outer myometrium terminating caudally in a muscular cervix. The volume (mm [3]) of the uteri (glandular portion and cervix), vaginal cul-de-sac, lateral vaginae, and urogenital sinus was calculated using the following equations. The glandular portion of each uterus was estimated as an ellipsoid, 4/3*π*(a/2*b/2*c/2); the cervices, lateral vaginae, and urogenital sinus as cylinders, π*r^2^*c; and the vaginal cul-de-sac as a cone, 1π*r^2^*c/3 (a = length, b = width, and c = depth). For cylindrical structures, average estimates of width and depth were calculated from the transverse measurements over the entire length of the structure. While vestibular glands were also identified opening into the caudal extremity of the urogenital sinus, no changes in the volume of this tissue were recorded.

### 2.3. Histology

After euthanasia, a thorough necropsy was performed, and tissues collected and processed for histological examination. The tissues were embedded in paraffin wax, and sections (5 µm) stained with hematoxylin and eosin (HE). The criterion for the identification of normal tissue was that the individual organ was histologically within normal limits, i.e., the absence of any histopathological changes, including degeneration, inflammatory reactions, and cell adaptations. The presence of pathology in the urinary tract was not considered an exclusion factor for the selection of a normal reproductive organ.

### 2.4. Phases of the Koala Reproductive Cycle

Johnston et al. [15,16] have identified 3 possible outcomes for the koala estrous cycle: (1) an anovulatory cycle during which proestrus leads to estrus and the development of a preovulatory follicle, which fails to ovulate because of the lack of coital-seminal induction of ovulation, and which is followed by follicular atresia and the subsequent development of a new wave of follicles; (2) ovulation of the pre-ovulatory follicle following coitus, with the formation of a corpus luteum (CL) and pregnancy; and (3) ovulation following coitus, formation of a CL, but failure of conception.

As the majority of koala cadavers were randomly presented to veterinary hospitals in this study, the precise stage of the reproductive cycle could not be determined [16]. Consequently, three presumptive phases of the reproductive cycle of the koala were arbitrarily recognized: (i) interestrous phase, (ii) proliferative phase, and (iii) luteal phase (incorporating early luteal, mid-, and post luteal). These three phases of the koala reproductive cycle were partly based upon ovarian activity, as indicated by various stages of follicular or luteal development, and the corresponding histological appearance of reproductive tract tissues.

The interestrous phase included the three states of anestrus, otherwise impossible to differentiate further in cadaver specimens: lactational anestrus (based on teat development but with no pouch young present), seasonal anestrus, and the interestrous period of a non-mated cycle. Ovaries in the interestrous phase were defined as containing Graafian follicles less than 3 mm in diameter but no grossly identifiable CL. The proliferative phase was identified here as the combined stages of pro-estrus and estrus, leading up to and immediately prior to ovulation. Graafian follicles of 3 to 4 mm in diameter that showed no gross increase in size of the reproductive tract (but had microscopic evidence of hyperplasia) were considered to be in an early proliferative stage, whereas females with ovaries containing Graafian follicles greater than 4 mm in diameter and with evidence of both hyperplasia of the mucosa and hypertrophy of the reproductive tract were considered to be in a late proliferative stage. For the purpose of analysis, these two groups were combined as representing the proliferative phase. The luteal phase was represented by the presence of a large corpus luteum. Although the non-pregnant state of the luteal phase was the prominent category analyzed in this study and was categorized by the absence of a conceptus in an otherwise enlarged uterus, there were 2 confirmed pregnant koalas for which data were also obtained. If the koala contained a CL but showed evidence of endometrial degeneration, then it was classified as being in a post-luteal condition and also grouped in the luteal phase category for the gross anatomy study. In addition, the reproductive histology of 2 koalas confirmed to be in the early stages of their post-luteal phase were examined. Although it was difficult to differentiate the latter stages of the post-luteal phase from the subsequent interestrous period without direct reference to corresponding endocrinological or behavioral data, the details of CL regression and post-luteal histological changes to the koala reproductive tract for which the ages of the CLs were accurately known, were available from 3 koalas that died in captivity; however, information from these koalas was not included in the gross anatomy study.

The studies of volume change of the gross anatomy study were based on the data of Johnston [12] and consisted of 50 reproductive tracts: interestrous (*n* = 34), proliferative (*n* = 8), and luteal (*n* = 8). The majority of the histological observations of the reproductive system were described from 20 animals processed from Pagliarani [13] (interestrous (*n* = 9), proliferative (*n* = 7), and luteal (*n* = 4)), but were further supported by the addition of ovaries and reproductive tracts also detailed in Johnston [12].

### 2.5. Statistical Analysis

Changes in the size and/or volume of the ovaries, the glandular portion of the uteri, cervices, vaginal cul-de-sac, lateral vaginae, urogenital sinuses, and clitoris were compared via a series of 1 way analysis of variance with respect to the three phases of the reproductive cycle using Statview SE + Graphics (Abacus Concepts, Berkley, CA, USA) and GraphPad Prism 8.0.1.244 (Dotmatics). The level of statistical significance was set *p* < 0.05.

## 3. Results

### 3.1. Gross Anatomy

**Figure 2:**
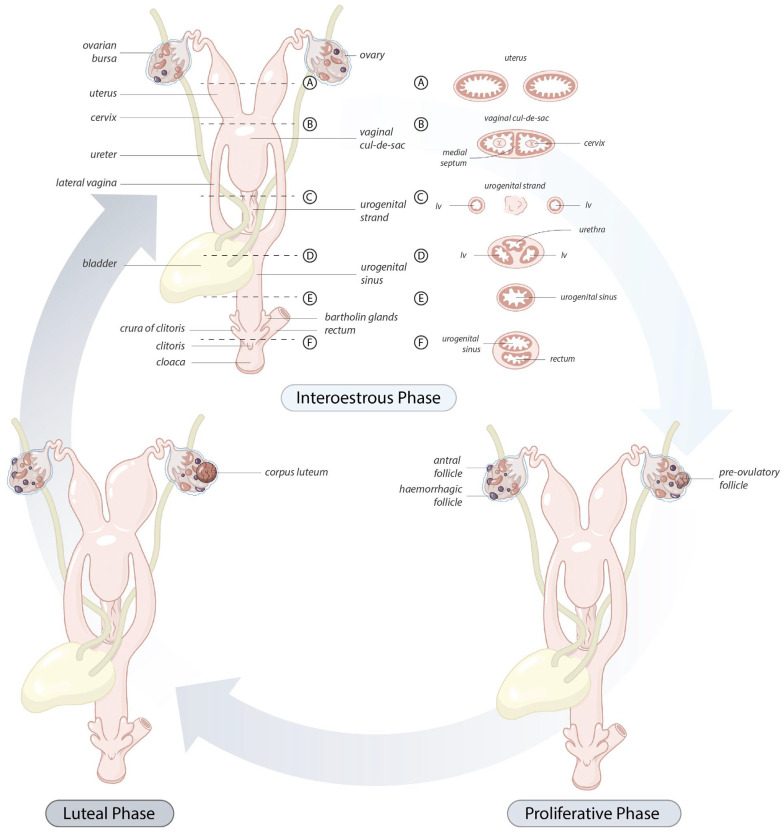
Schematic drawing of an example of the female koala reproductive system in the different stages of the reproductive cycle showing the relative cross-sectional sections and changes in volume of the respective components. lv—lateral vagina.

In the female koala reproductive tract, the two ovaries are each suspended within the peritoneal bursae attached to the dorsal abdominal wall (Figure 1 and Figure 2 [see inset]). The ovary was partially covered by a fimbriated infundibulum, which subsequently extended into a glandular ampulla and muscular isthmus. Each oviduct attached to a glandular uterus that extended to a well-developed muscular cervix. Each cervix opened into a medially partitioned vaginal cul-de-sac complex, both of which received the terminal cranial portion of a muscular lateral vagina. The external caudal ostium of each lateral vagina appeared to arise from the distinctive mucosal folds of the urogenital sinus that exited via the cloaca, ventral to the rectum. The relative positions of the entrances of the lateral vaginae into the upper vaginal cul-de-sac varied between individual koalas; some vaginae opened into the vaginal cul-de-sac immediately caudal to the entrance of the cervix. In other koalas, the vaginae opened more caudally from the cervical ostia. The lateral vaginae of interestrous koalas were slightly curved longitudinally (Figure 2), and essentially circular in transverse section throughout their length. The caudal extremities (first 2 to 3 mm) of the lateral vaginae were thin-walled, less muscular, and more dilated than the remainder of the vaginae.

While ovarian bursae, ovarian vasculature, oviducts, uteri, and the cranial portion of the vaginal complex were readily identified on laparotomy after diversion of the colon and caecum, the lateral vaginae, lower vaginal cul-de-sac, and urogenital sinus were hidden within the pelvic canal, and in some specimens encircled by a thick (up to 7 mm) layer of abdominal fat; this fat layer was often the only visibly detectable adipose tissue in the koala abdomen. The lower portion of the reproductive tract could only be accessed after pelvic floor resection (Figure 1).

A careful dissection of the tissue around the most caudal portion of the urogenital sinus revealed the presence of a pair of bilaterally arranged vestibular or Bartholin’s glands (Figure 2). These structures were located parallel with the ventral aspect of the caudal urogenital sinus, embedded in connective tissue, and sandwiched between an outer layer of circular muscle and the muscularis of the urogenital sinus. The gross appearance of these structures varied between individual koalas. In two females, the vestibular glands were heart-shaped; however, in the third female examined, the gland was a simple cylindrical structure approximately 10 mm in length and 3 mm in diameter. Each vestibular gland was drained by an excretory duct which emptied into the urogenital lumen via a small papilla-like opening located on the rim of a ventral depression in the caudal extremity of the urogenital sinus. In close proximity to the vestibular glands, but located slightly caudolaterally, were the bilaterally arranged crura of the clitoris. Protruding from the ventral floor of the urogenital sinus into the common vestibule was a dorso-ventrally flattened bifid clitoris. The lineal dimensions of the clitoris are reported in Table 1. In the interestrous koala, the length of the common vestibule from the outer edge of the vestibular lip to the start of the urogenital lumen was 8.7 ± 0.3 mm.

### 3.2. Gross Anatomical Changes of the Koala Reproductive Tract throughout Different Phases of the Reproductive Cycle

Table 1 documents the lineal dimensions and/or relative changes in the volume of different components of the reproductive tract with respect to the different stage of reproductive cycle (interestrous, proliferative, and luteal phases); these changes are also represented pictorially in Figure 2. The results of this analysis revealed significant differences in the volume of respective components of the koala reproductive tract in the three stages of the reproductive cycle as determined by the ovarian/reproductive tract activity. There was a progressive increase in the volume of the uterus from the interestrous to the proliferative stage (3.5×), and a further increase from the proliferative stage to the luteal phase (2.7×). When glandular uteri in all three of these phases of the reproductive cycle were analyzed together, there was no significant difference in volume between left or right uterus (t = 1.55; *p* = 0.1277), the only notable exceptions being two gravid uteri (7.1 mL and 6.4 mL), which were, respectively, 5.7 and 1.8 times larger in volume than their contralateral, non-gravid uteri. The volume of the cervices in the proliferative phase was greater than that of the interestrous and luteal phases. The volume of the vaginal cul-de-sac, lateral vaginae, and urogenital sinus was also greatest during the koala proliferative stage.

### 3.3. Ovarian and Reproductive Tract Histology of the Koala Interestrous Phase

**Figure 3:**
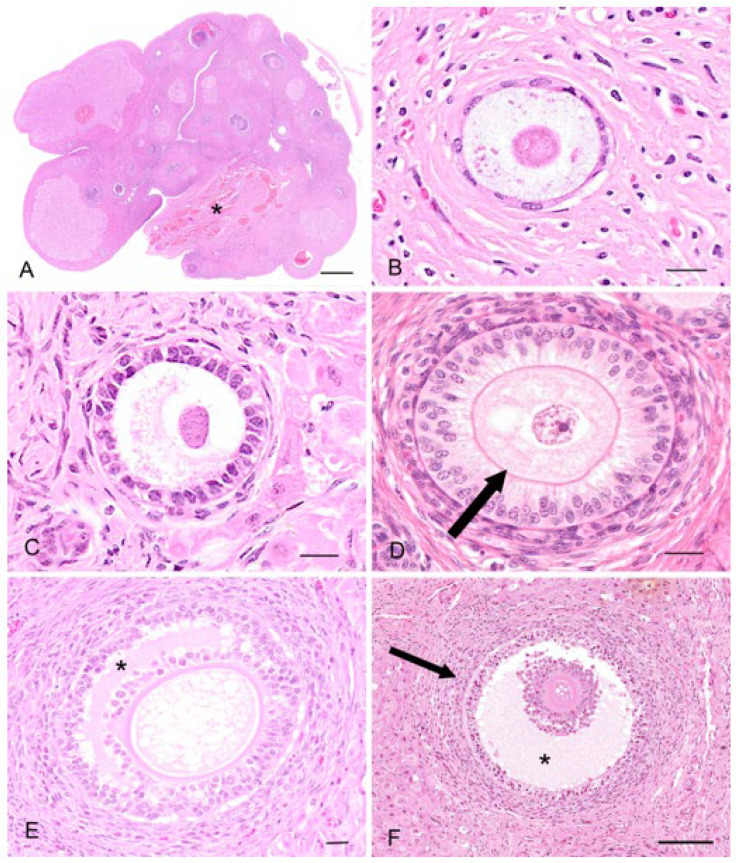
Histological features of the koala’s ovary in the interestrous phase. (**A**) Outer cortical and inner medullary region of the ovary, converging into the hilus containing numerous blood vessels (asterisk). HE. Bar = 1 mm. (**B**) Primordial follicle with an oogonium surrounded by a single layer of flat follicular cells. HE. Bar = 20 µm. (**C**) Primary follicle with a central oocyte surrounded by a single layer of cuboidal follicular cells. HE. Bar = 20 µm. (**D**) Secondary follicle with columnar follicular cells and the appearance of the zona pellucida (arrow). HE. Bar = 20 µm. (**E**) Early stage of antrum formation (asterisk) with multiple layers of follicular cells. HE. Bar = 20 µm. (**F**) Graafian follicle with theca cells (arrow) and a single cavity containing follicular fluid (asterisk). HE. Bar = 100 µm.

The koala reproductive tissue in the interestrous phase will be used here to give a general base-line histological description of the ovary and reproductive tract, from which to compare relative changes in histology accompanying the proliferative and luteal phases. The ovaries of the interestrous koala were typically dorso-ventrally flattened ellipsoidal structures and on their surface typically contained one or more Graafian follicles of 3 mm or less in diameter. The koala ovary can be arbitrarily divided into an outer cortical and an inner medullary region, which merged with the vascular connective tissue of the mesovarium at the hilus of the ovary (Figure 3A). The medulla includes nerves, large blood vessels and lymphatic vessels entering the ovary from the mesovarium and through the hilum. Arteries entered the ovary at the hilum, and in the medulla they formed plexuses, giving rise to small arterial branches to the theca cells of the follicles, corpora lutea, and the stroma cells. The venous return was parallel to the arterial supply. The nerves that supply the ovary appeared to be non-myelinated, followed blood vessels, and terminated in the walls of the vessels and around the follicles, in the corpora lutea (luteal phase), and in the tunica albuginea. The cortex consisted of ovarian follicles at different stages of development embedded within stromal connective tissue. The outer cortex was covered by a superficial mesothelium derived from the visceral peritoneum consisting of single flat or cuboidal (5–8 µm in diameter) epithelial cells. The nuclei of the superficial epithelium contained round to oval hyperchromatic nuclei (2–3 µm in diameter) with dispersed chromatin. Immediately beneath the superficial epithelium lay a thick layer of connective tissue (60–70 µm in width), the tunica albuginea.

Primordial follicles measured 30–40 µm in diameter and consisted of an oogonium of approximately 7–10 µm diameter, surrounded by a single layer of flattened epithelial-like follicular cells (simple squamous epithelium; Figure 3B). A thin basal lamina separated the follicle from the ovarian stroma. The primordial follicles were mostly located adjacent to the outer extremity of the cortex and could be found in groups of 5–10 follicles. In the early stages of oocyte growth, follicular cells surrounding the oocyte changed from flattened to cuboidal in appearance to denote the formation of a primary follicle (Figure 3C). The primary follicles (70–80 µm in diameter) consisted of a primary oocyte encircled by a single layer of now cuboidal follicular cells. By the time the second layer of follicular cells was formed around the oocyte (secondary follicle), the follicular cells were essentially columnar in appearance, and the zona pellucida discernible as a thin eosinophilic layer around the circumference of the oogonium (Figure 3D). The oocyte of the secondary follicle was encircled by a stratified epithelium of follicular polyhedral granulosa cells. These cells had a round to elongated nucleus, and were occasionally hyperchromatic, but otherwise vesicular with dispersed chromatin and a moderate amount of eosinophilic cytoplasm. The oocyte was characterized by a well-defined round nucleus with a round central to eccentric nucleolus and granular chromatin. Thecal tissue was also clearly delineated into external and internal layers by the time the secondary follicle was 130 µm in diameter. The cells forming the theca interna layer had an elongated nucleus with a scarce amount of eosinophilic cytoplasm.

Early stages of antrum formation occurred when the diameters of the follicle and oocyte were approximately 230 µm and 120 µm, respectively. The antrum formation coincided with the appearance of the third and/or fourth layer of follicular cells (Figure 3E). The antrum formation was characterized by the coalescence of small fluid-filled fissures to form a single cavity containing the follicular fluid (liquor folliculi). The typical koala *Graafian follicle* (2 to 3 mm) (Figure 3F) of the interestrous ovary consisted of an outer theca externa composed of elongated fibrous cells, measuring 42.6 ± 2.7 µm (*n* = 51) thick. The outer extremity of the theca externa was often difficult to clearly delineate from the surrounding stromal tissue. The theca interna measured 41.9 ± 2.2 µm (*n* = 51) and consisted of slightly enlarged epithelioid stromal cells. Both theca interna and theca externa were supplied with numerous blood vessels. The koala oocyte was usually suspended in the antrum to one side of the fluid-filled follicle by a discus proligerus. A basement membrane separated the cells and blood vessels of the theca from the granulosa cells. The thickness of the zona pellucida, measured from 10 oocytes dissected free from 3 mm follicles, was 14.2 ± 0.6 µm. Incidental histological sections of ovaries from two 6-month-old and two 10-month-old pouch young collected opportunistically also revealed the presence of Graafian follicles. In all koala ovaries examined, at least one Graafian follicle was found per ovary, independent of the koala’s body condition, time of the year the ovary was recovered, or whether or not the koala was lactating.

**Figure 4:**
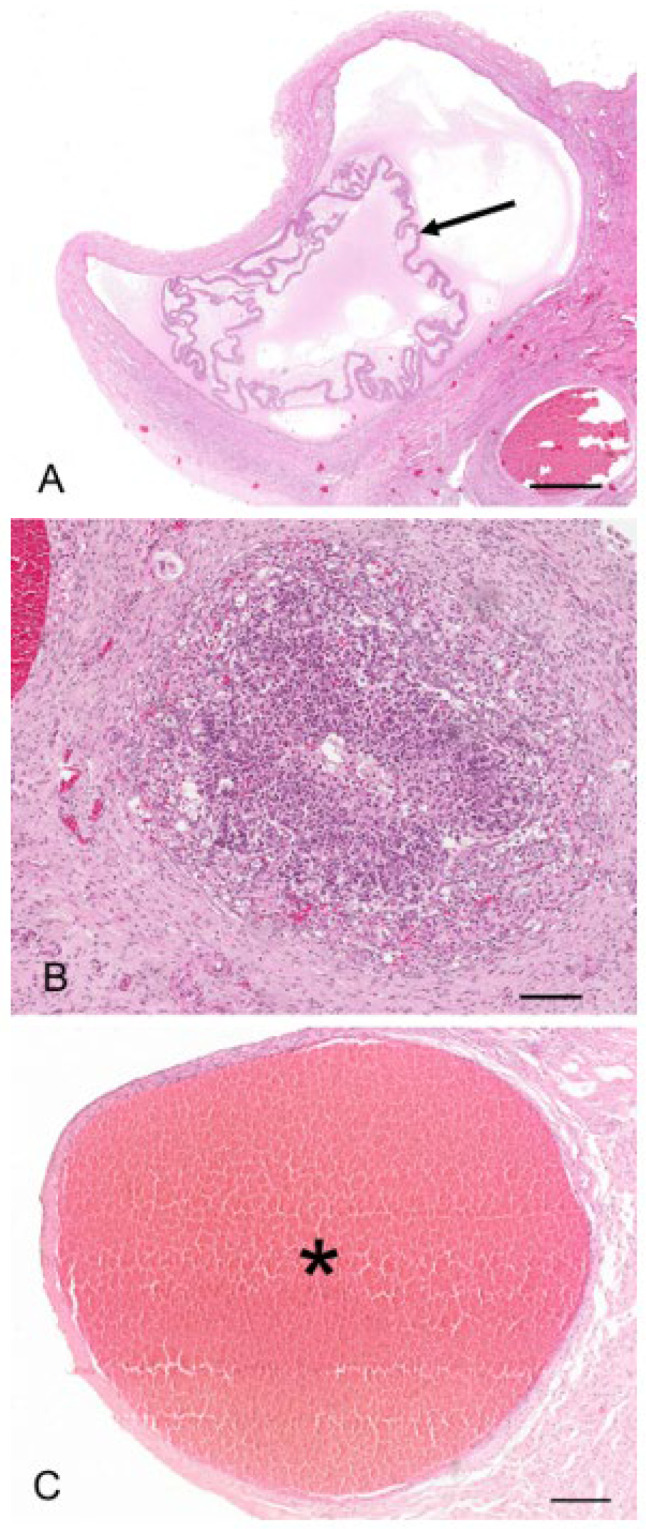
Histological features of follicular atresia (**A**,**B**) and hemorrhagic follicles (**C**) in the koala’s ovary. (**A**) Dilated atretic follicles with detachment and irregularity of the stratum granulosum (arrow). HE. Bar = 700 µm. (**B**) Obliterative atresia with invasion of the antral space by hypertrophic granulosa and theca cells. HE. Bar = 100 µm. (**C**) Hemorrhagic follicle consisting of a large blood-filled cavity (asterisk). HE. Bar = 100 µm.

The regulation of the number of follicles in the developing follicular pool includes a normal process known as follicular atresia. The main features of atresia in follicular and granulosa cells are pyknosis and chromatolysis of the nucleus. During atresia, the basal membrane of the stratum granulosum can become thicker, more translucent, and irregular (membrana vitrea) (Figure 4A). When atretic follicles are reabsorbed, small scarring areas of fibrotic tissue are left. Both types of atresia, obliterative atresia and cystic atresia, were noted in the koala ovary. In obliterative atresia, the granulosa and theca layers were irregular, hypertrophic, and occupied the entire antral space. Hypertrophic cells were spindle-like cells, 5–15 µm in diameter, with clear eosinophilic cytoplasm (Figure 4B). During cystic atresia, the granulosa layer became atrophic while the theca cells started to develop luteinization features, such as an enlarged polygonal shape, ample eosinophilic cytoplasm, and the presence of scattered lipid droplets.

A typical feature of the koala ovary, but more so in the interestrous phase, was the presence of hemorrhagic follicles (Figure 4C). The majority of histological sections taken through these structures revealed a thin theca externa encapsulating a blood-filled cavity. Less commonly, blood-filled cavities were also surrounded by a theca interna or membrana granulosa, the cells of which were in various stages of hyperplasia.

**Figure 5:**
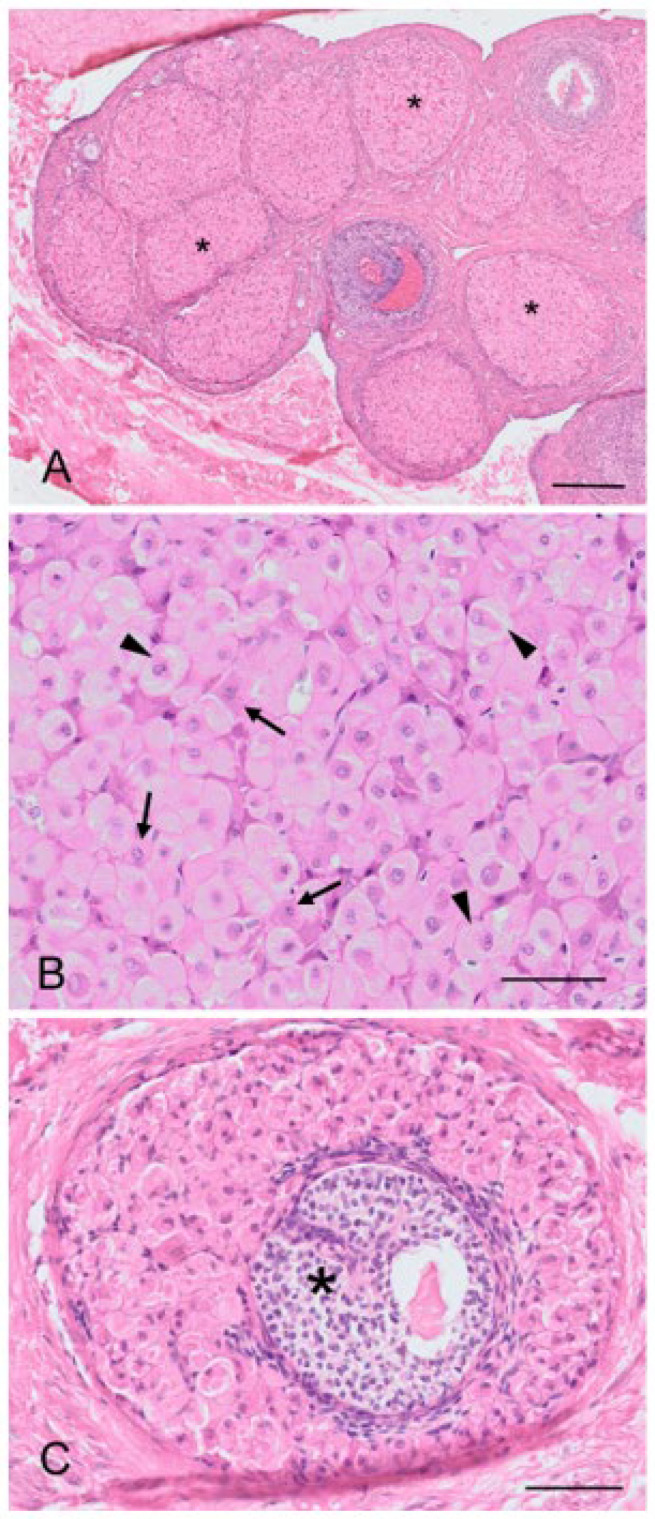
Histological features of the interstitial-like tissue within the koala’s ovary. (**A**) Multiple aggregates of interstitial-like tissues occupying 80–90% of the ovary (asterisks). HE. Bar = 200 µm. (**B**) Type I cells with pales eosinophilic often vacuolated cytoplasm (arrowheads) and Type II cells with strong eosinophilic cytoplasm (arrows). HE. Bar = 50 µm. (**C**) Residual follicle with multiple layers of granulosa cells (asterisk) encircled by the interstitial-like tissue. HE. Bar = 50 µm.

The cortex of most koala interestrous ovaries examined contained discrete foci of “interstitial-like” tissue encapsulated by layers of connective tissue (Figure 5A). Apart from connective tissue cells and a blood supply, this “interstitial-like” tissue was composed of at least two cell types which were distinguished by their relative size and degree of cytoplasmic eosinophilia (Figure 5B). Type “I” cells (28–43 µm in diameter) are described as ovoid with a slightly eosinophilic cytoplasm. The nucleus was large (7–8 µm in diameter and occupying 1/3 of the cell cytoplasm), and round with finely dispersed chromatin. These cells were large and often polyhedral in shape, their cytoplasm containing lipid droplets, thus having the histological appearance of steroidogenic cells. Type “II” cells (13–26 µm in diameter) were polygonal, spindle-like, and contained a strongly eosinophilic cytoplasm; the nucleus (3–7 µm in diameter) of these cells was round and had mostly condensed central chromatin. Every aggregate of interstitial cells was surrounded by connective tissue admixed with reticular fibers. From the observation of numerous atretic follicles in various stages of degeneration, it was apparent that the cells of the theca interna, and possibly in some instances, membrana granulosa, may have been the source of this “interstitial-like” tissue (Figure 5C). Interestingly, no tissue resembling “interstitial-like” tissue was found in the ovaries of a 6-month-old pouch young, but numerous atretic follicles showed evidence of hyperplasia.

The koala ovary was enclosed within a thin peritoneal *ovarian bursa* which was composed of three layers, the bursal epithelium facing the ovary, a connective tissue layer, and the peritoneal mesothelium (Figure 2 and [13]). The middle layer contained connective tissue with fibroblasts, bundles of smooth muscle cells, and blood vessels. The bursal epithelium of the koala was a continuous layer of cells resting on a dense mat of collagen fibers. The epithelial cells (12–14 µm in diameter) varied in shape from cuboidal to elongated. The nuclei (1–2 µm in diameter) of cuboidal cells were ovoid, whereas in elongated cells they were more cigar-shaped and smooth. The cell surface was covered with microvilli of a fairly uniform length. The peritoneal surface was covered by a continuous layer of ciliated and non-ciliated mesothelial cells (12–14 µm in diameter), resting on a basal lamina. The nuclei (1–2 µm in diameter) of these cells were also elongated, narrow, and had a smooth outline. On the cranio-medial aspect of the ovarian bursa, there was a peritoneal opening which accommodated the passage of the cranial portion of the oviduct (infundibulum) and ovarian blood supply. As the histology of the bursa did not change with respect to the stage of the estrous cycle, it will not be described in further phases.

**Figure 6:**
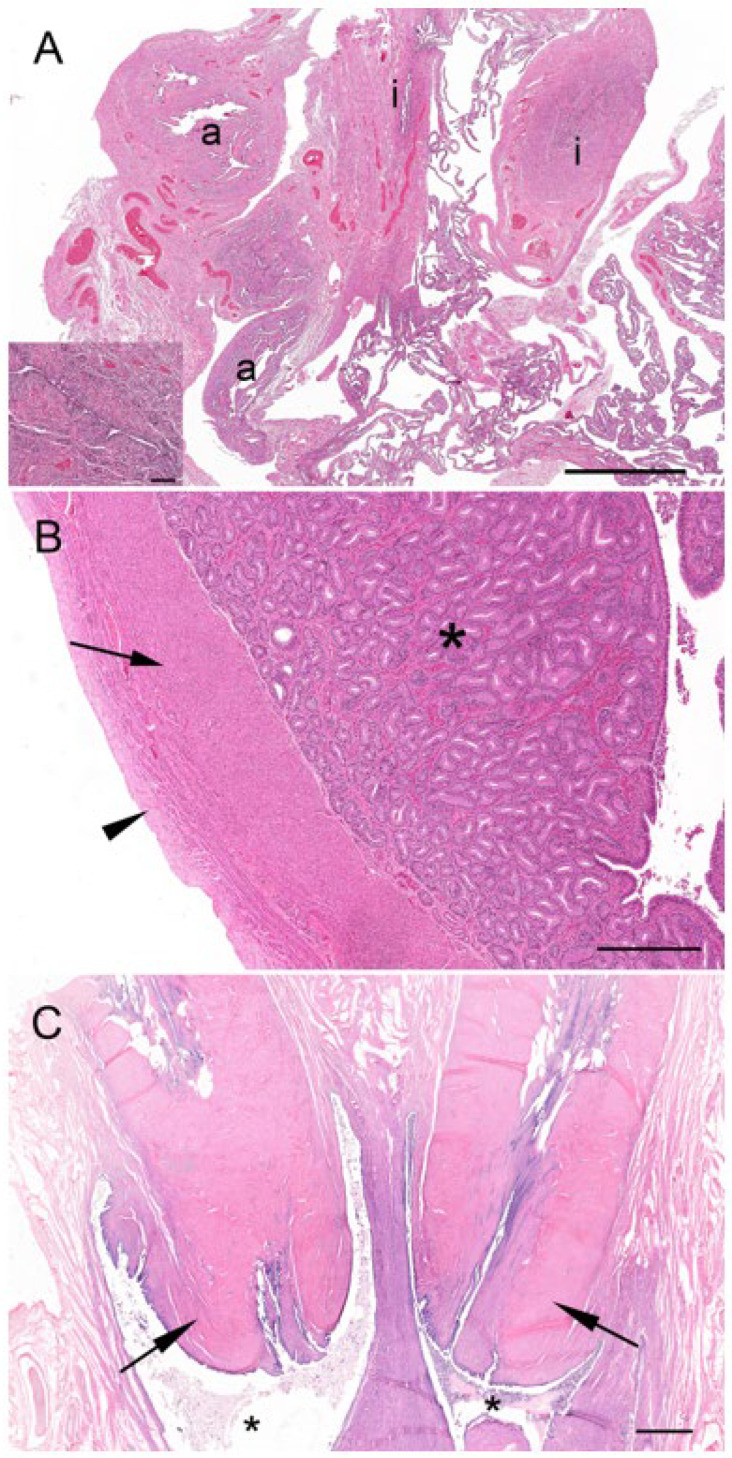
Oviduct (**A**), uterus (**B**), and cervix (**C**) of the female koala in the interestrous phase. (**A**) Ampulla (a) and isthmus (i) of the oviduct (inset: reduced number of the mucosal folds in the isthmus close to the uterine junction). HE. Bar = 2 mm (inset—Bar = 100 µm). (**B**). Uterine wall with mucosa/submucosa (asterisk), myometrium (arrow), and serosa/perimetrium (arrowhead). HE. Bar = 600 µm. (**C**) Protrusion of the caudal extremities of the muscular cervices (arrows) into the corresponding vaginal cul-de-sac complex (asterisks). HE. Bar = 1 mm.

The koala oviduct was a tortuous tubular organ extending adjacent to the ovaries within the bursa to the uterine horns. Each oviduct could be divided into three distinctive sections, the infundibulum, an initial large funnel-shaped portion, the ampulla (Figure 6A), a thinner section extending caudally from the infundibulum, and the isthmus, a narrow muscular segment connecting to each uterus. The epithelium of all sections was flattened to simple cuboidal (12–14 µm in diameter), with the majority of cells, especially those of the infundibulum, possessing mobile cilia (and some microvilli). The nucleus (5–8 µm in diameter) of these epithelial cells was mostly eccentric, and round with condensed chromatin. A few cup-like cells, goblet cells, were present amongst the epithelial cells of the infundibulum; these cells were simple columnar epithelial cells (10 µm in diameter), having a height of four times their width. The cytoplasm was displaced toward the basal end of the cell body by large mucin granules accumulating at the apical surface of the cell. The mostly basophilic basal part of the cell contained a large round to oval nucleus (4–5 µm in diameter). The oviduct submucosa was composed of loose connective tissue rich in plasma cells, eosinophils, and a few mast cells. The mucosal-submucosal layer of the koala ampulla consisted of several longitudinal folds, gradually reducing in size and number closer to the isthmus-uterine junction (Figure 6A insert). While the ampullary epithelium contained numerous goblet cells (secretory epithelium), ciliated cells were not common in the oviduct of the interestrous koala. The external diameter of the isthmus increased proximally as it approached the utero-tubal junction. The lumen of this region was thrown into five to six longitudinal folds of a simple cuboidal epithelium. The outer diameter of the isthmus increased as it looped towards the uterus, which appeared to be due to a corresponding increase in thickness of the muscularis and outer connective tissue.

The wall of the uterus was composed of three layers, the mucosa-submucosa or endometrium, the myometrium, and the serosal layer or perimetrium (Figure 6B). The endometrium consisted of two sections, being distinctive by their structure and function: a superficial layer or “functional zone, and a thin deeper layer or “basal zone”. The lining epithelium of the functional zone was simple columnar in the koala (15–20 µm in diameter), with an oval to round nucleus (4–5 µm in diameter) and condensed chromatin. The columnar epithelium included secreting cells and ciliated non-secreting cells. The myometrium consisted of a circular inner thicker layer (800 µm in diameter) and a longitudinal layer of smooth muscular cells (250 µm in diameter); in-between these layers, a vascular area composed of large arteries, veins, and lymphatic vessels was present. The perimetrium, or *tunica serosa*, of the uterus consisted of loose connective tissue covered by the peritoneal mesothelium; it included smooth muscular cells, vessels, and nerves. Interestrous females had small uteri with a narrow uterine endometrium with few folds, and short and relatively narrow glands with small lumens that occupied a small percentage area of the endometrium. The endometrial epithelium of interestrous koalas was composed of tall columnar cells and scattered polymorphonuclear cells infiltrated the lamina propria. The uterine glands in this phase were sparse and relatively small (diameter 70–80 µm). The endometrial epithelium of the sections showed scattered areas of intracytoplasmic vacuolar degeneration, and low numbers of mitotic figures were generally observed in the luminal uterine epithelium. The epithelial cells in the endometrial lumen had scattered ciliated cells. While the uteri of females in lactational anestrus were similar in size to those in other stages of interestrus, they also possessed non-ciliated uterine and glandular epithelial cells with irregular nuclei, with large amounts of degenerated cells (leukocytes) accumulated in the uterine gland lumen; mitotic activity was also noted more frequently in the uterine epithelial cells of these animals.

The caudal extremities of both muscular cervices protruded into their respective compartments of the vaginal cul-de-sac complex (Figure 6C). The longitudinal and transverse sections of the cervices revealed the lumen to be narrow and convoluted. The columnar epithelium lining the lumen of the cervices was arranged in shallow longitudinal folds, lined by a single layer of cuboidal epithelium (15–20 µm in diameter), with several muciparous cells, including goblet-shaped cells scattered amongst them; the secreting cells in the cervix were similar to those described in the oviductal infundibulum. The epithelial cells were characterized by an oval nucleus (5 µm in diameter) with condensed chromatin. The propria-submucosa consisted of thick irregular connective tissue, subject to changes in its thickness during the different phases of the cycle. The tunica muscularis consisted of an inner circular layer, rich in elastic fibers, and an external layer of smooth muscle cells. The tunica serosa of the cervix consisted of loose connective tissue and lining mesothelial cells.

**Figure 7:**
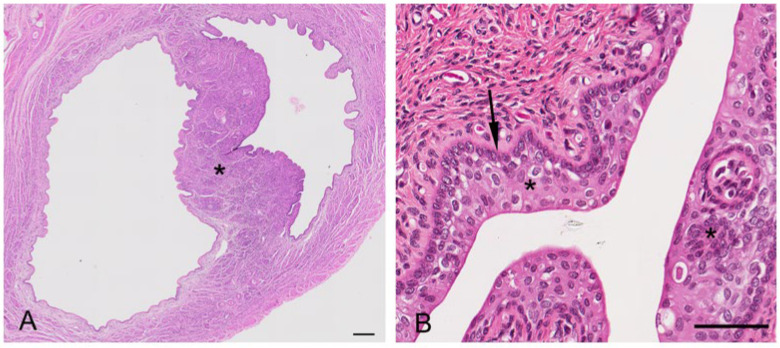
Vaginal cul-de-sac (**A**) and lateral vagina (**B**) of the female koala in the interestrous phase. (**A**) Medial septum (asterisk) partitioning the vaginal cul-de-sac. HE. Bar = 200 µm. (**B**) Stratum basale (arrow) and stratum spinosum (asterisk) lining the lumen of the lateral vaginae. HE. Bar = 60 µm.

The vaginal cul-de-sac of the interestrous koala was lined by a simple cuboidal epithelium (10–15 µm in diameter), with a round to oval regular nucleus (5–6 µm in diameter) and condensed chromatin, as was the medial septum partitioning the vaginal cul-de-sac (Figure 7A). The basal lamina of the cul-de-sac containing loose connective tissue separated the epithelium from the submucosa, consisting of connective tissue, small blood vessels, and a large amount of lymphoid tissue. Scattered polymorphonuclear cells were also observed in the submucosa during the interestrous stage. Longitudinally arranged smooth muscle cells were present in the highly vascular muscular layer (*tunica muscularis).* In addition to the connective tissue and vascular structure, the submucosa of the medial septum was also composed of several layers of longitudinally arranged muscle fibers. No patent birth canal or opening was observed between the caudal extremities of the cul-de-sac and the urogenital sinus in any of the interestrous reproductive tracts examined.

The lateral vaginae of the koala were two tubular muscular organs, extending from the cervices to the common urogenital sinus, lined by flat longitudinal folds of the mucosa and submucosa layers. The lumina of koala lateral vaginae featured 6 to 11 longitudinal folds and were lined for almost their entire length by a stratified squamous epithelium (5–8 µm length). The deeper cellular layer, the stratum basale, consisted of a single sheet of cuboidal cells (6–8 µm in diameter), with an apical nucleus containing uniform condensed chromatin (Figure 7B); and a stratum spinosum, made of a variable number of highly interconnected polyhedral cell (5–8 µm in diameter). As these cells reach the outer layers, they become flatter. This specific layer is then called stratum granulosum, which is not present when the epithelium is non-cornified. The propria-submucosa consisted of loose or irregular connective tissue, containing lymphatic nodules in the caudal section of the vagina. The tunica muscularis was made of two or three layers of smooth muscular tissue; the innermost layer was thickened, separated by connective tissue and covered by a thin external longitudinal layer. The tunica adventitia (or tunica serosa more cranially) consisted of loose connective tissue and major vessels, nerve bundles, and ganglia.

**Figure 8:**
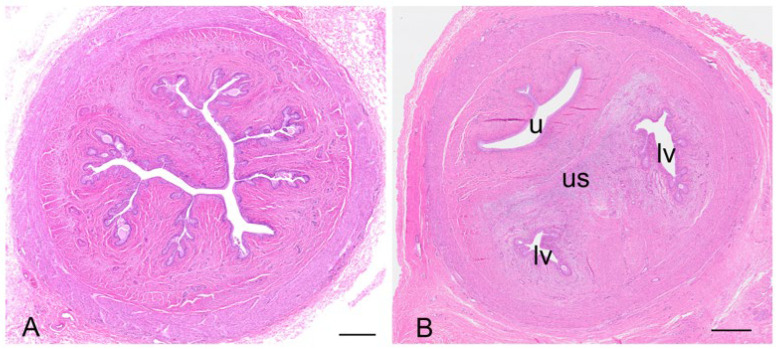
Urogenital sinus (**A**) and urogenital strand (**B**) of female koala in the interestrous phase. (**A**) Prominent longitudinal mucosal folds lining the lumen of the urogenital sinus. HE. Bar = 500 µm. (**B**) Urogenital strand (us) positioned between the urethra (u) and lateral vaginae (lv), and consisting of connective tissue, smooth muscle fibers, and blood vessels. HE. Bar = 500 µm.

The urogenital sinus of the interestrous koala was widest at its cranial and caudal extremities, in association with distinct swellings at the junction of the canal with the lateral vaginae and urethra (‘urogenital swelling’) and the opening of the canal into the common vestibule, respectively. The most characteristic feature of the urogenital sinus was the presence of 9 to 10 prominent longitudinal mucosal folds which ran over the length of the canal (Figure 8A). These folds of tissue appeared to define a pathway cranially, to the point where they ultimately coalesced to join the distinctive lumina of the lateral vaginae and urethra. The mucosal folds observed throughout the entire luminal surface of the urogenital sinus were lined with four/five layers of stratified non-cornified/cornified squamous epithelium (each cell 6–9 µm in diameter), with only occasional degenerated cells, showing hydropic changes and pyknotic nuclei.

The connective tissue between the caudal extremity of the vaginal cul-de-sac and the cranial limit of the urogenital sinus is known as the urogenital strand. The urogenital strand begins in the region where the lateral vaginae and urethra coalesce with the longitudinal folds of the urogenital sinus and terminates at the caudal extremity of the vaginal cul-de-sac. The strand consisted of a section of tissue positioned between the surrounding musculature of the lateral vaginae, urethra, and outer muscularis of the urogenital sinus, and was composed of densely packed connective tissue, smooth muscle fibers, and blood vessels (Figure 8B).

Due to the high variability in the gross appearance and location of the vestibular glands (Figure 2), only two pairs of vestibular glands during the interestrous phase were examined. The tubulo-acinar glandular component was lined by a layer of basal cells beneath the columnar epithelium. The basal cells were flat to cuboidal, 7–10 µm in diameter, with scarce to moderate cytoplasm, a central vesicular round nucleus, and granular chromatin. The columnar epithelial cells were approximately 7 µm wide and 20 µm long, with a central to paracentral nucleus and abundant eosinophilic cytoplasm. Scattered degenerated epithelial cells were described in the glands (pyknotic nuclei, hydropic degeneration). The glandular lumen did not contain any secretory material, only scattered leukocytes and cell debris in one specimen, and highly basophilic secretory material with a few leukocytes in the other. The superficial fibrous capsule was continuous with an extensive stroma internally supporting lobules of secretory parenchyma. Each vestibular gland was drained by an excretory duct lined by a single layer of cuboidal epithelial cells, which emptied into the urogenital lumen via a small papilla-like opening in the caudal extremity of the urogenital canal.

Protruding from the ventral floor of the koala urogenital sinus into the common vestibule was the clitoris. The koala clitoris was a dorso-ventrally flattened bifid structure contained within the walls of the vestibule. It consisted of broad longitudinal folds of the mucous membrane, lined by a stratified non-cornified squamous epithelium. The supporting stroma was made of loose connective tissue. The clitoris consisted of several lymphatic nodules and nerve ganglia.

### 3.4. Ovarian and Reproductive Tract Histology of the Koala Proliferative Phase

**Figure 9:**
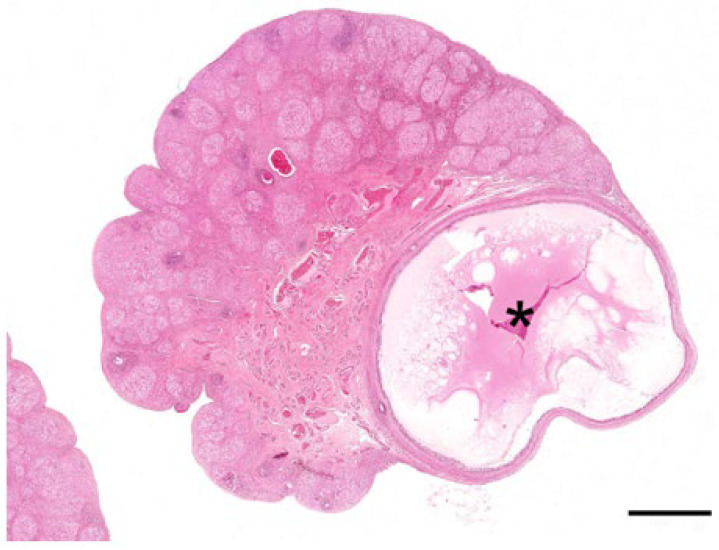
Histological features of the koala’s ovary in the proliferative phase. Large tertiary follicle (asterisk) in the ovarian cortex. HE. Bar = 2 mm.

The main feature of the koala ovary in the proliferative phase was the presence of a large tertiary follicle in the ovarian cortex (Figure 9). Protruding from the surface of the ovary, the presumptive pre-ovulatory follicle in the koala was remarkably large, and in one koala measured 6.8 mm in diameter. This large fluid-filled structure consisted of a theca externa, a theca interna composed of one to two cell layers, and a granulosa cell layer of 5 to 8 cells thickness. The primary oocyte in the tertiary follicle of the koala was 110–130 µm in diameter, spheroidal, and contained a central nucleus with condensed chromatin and a thick zona pellucida (6–8 µm in diameter). As the antrum enlarged, the oocyte became eccentric, and two or three layers of granulosa cells (30–40 µm in diameter) called the cumulus oophorus surrounded the oocyte. Polyhedral granulosa cells formed the internal layer of the follicle which is known as the stratum granulosum (granulosa layer). Diffuse papillary hyperplasia was also observed on the superficial epithelium of the ovary during the proliferative phase of the cycle in several koala specimens. Oocytes recovered directly from two 6 mm follicles measured 166 and 167 μm in diameter, while the zonae pellucidae of these oocytes were 14.4 and 15.6 μm thick, respectively; no polar bodies or metaphase plates were observed in either oocyte.

The oviducts of the koalas in the proliferative phase of the reproductive cycle showed marked changes in the histological appearance of the epithelium. The mucosa of the isthmus was highly convoluted, and the epithelium consisted of a single layer of hyperplastic columnar cells. Similarly, the ampullary region showed hyperplastic changes resembling those of the isthmus. The epithelial lining of the ampulla contained numerous goblet and ciliated cells.

**Figure 10:**
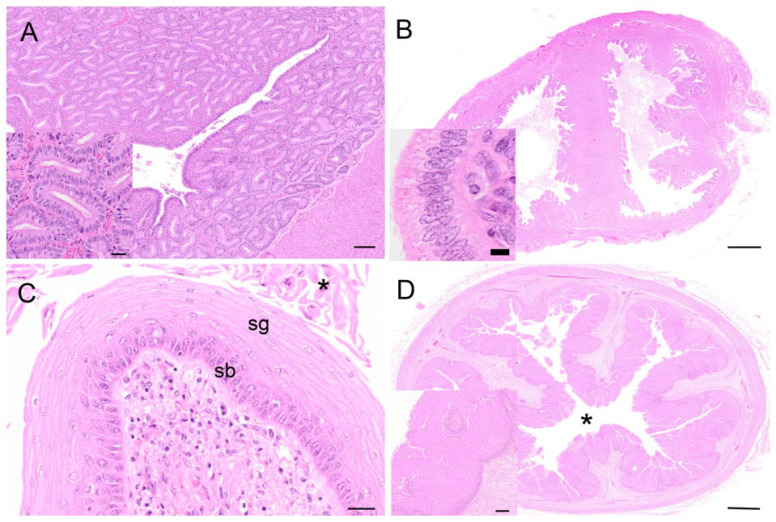
Histological features of the uterus (**A**), vaginal cul-de-sac (**B**), vagina (**C**), and urogenital sinus (**D**) of the koala’s ovary in the proliferative phase. (**A**) High number of glandular structures within the mucosa/submucosa of the uterus (inset: glandular tissue lined by columnar epithelial cells). HE. Bar = 200 µm (inset—Bar = 20 µm). (**B**) Increased folding of the epithelium lining the lumen of the vaginal cul-de-sac with cells transitioning to a columnar epithelium (inset). HE. Bar = 1 mm (inset—Bar = 5 µm). (**C**) Formation of the stratum granulosum (sg) over the stratum basale (sb) in the vaginal epithelium; note the desquamation of cornified epithelial cells in the lumen (asterisk). HE. Bar = 20 µm. (**D**) Hyperplastic and hypertrophic epithelial layer lining the lumen of the urogenital sinus (asterisk). Note the increased number of cells in the inset. HE. Bar = 1 mm (inset—Bar = 100 µm).

The endometrium of the koalas in the proliferative phase of the cycle consisted of a highly vascular submucosa, tightly packed with glandular tissue (Figure 10A). The lumen of the uterus was lined by a simple epithelium of cuboidal cells with occasional goblet cells. The glandular lumina were open and lined with simple hyperplastic ciliated columnar epithelium cells. Diffuse stromal oedema, ranging from mild to marked, was also observed. The epithelial cells were about 32 µm in diameter, with eosinophilic cytoplasm, and contained basally located nuclei with condensed chromatin. The koala proliferative uteri were characterized by mitoses apparent in both the uterine and glandular epithelium. Invaginations in the endometrial lining were also observed in this phase. The myometrium was unremarkable, about 1 mm thick, and composed of elongated muscle fibers.

The histology of the cervix from females in the proliferative phase was similar to that of the interestrous or luteal phases. The propria-submucosa, made of thick irregular connective tissue, was the only section subjected to changes in its thickness (increased) during the proliferative phase of the cycle.

During the proliferative phase of the koala reproductive cycle, there was an increase in the degree of folding of the epithelium over the surface of the medial septum and of the upper regions of the vaginal cul-de-sac adjacent to the cervical ostia (Figure 10B). The vaginal cul-de-sac epithelium was characterized by increased hyperplasia, with the cells transitioning from a cuboidal to a columnar appearance. The cul-de-sac was filled with an eosinophilic mucoid substance mixed with cellular debris and a few leukocytes. Several mitotic figures were detected within the epithelial layer, and little if any degeneration or desquamation was observed. Very few polymorphonuclear cells were described. Marked folding and hyperplasia of the luminal epithelium was observed during this phase.

The vaginal epithelium during the proliferative phase marked the formation of the stratum granulosum over the stratum germinativum (stratum basale) of the epithelium (Figure 10C). This consisted of flattened epithelial cells (15–20 µm in diameter), containing many keratohyalin granules within their cytoplasm and no nucleus. The formation of a stratum corneum of dense, cornified cells also characterized this phase. Some specimens, likely at the end of the proliferative stage, showed a fully cornified epithelium exhibiting some desquamation of superficial cells. Occasional scattered stromal leukocytic infiltrates were observed. In the cornified layers, if present, the superficial cells (stratum corneum) either had a pyknotic nucleus or lacked one (and possessed a high amount of keratin), while in the non-cornified epithelium those cells possessed a nucleus. The propria-submucosa consisted of a thinner layer of loose connective tissue, muscle fibers, and blood vessels compared to the lateral vaginae. The tunica muscularis consisted of two or three layers of smooth muscle fibers. The outer tunica adventitia consisted of loose connective tissue and large vessels.

Hypertrophy and hyperplasia of the epithelial layer of the urogenital sinus, with formation of the stratum corneum (8–10 µm in diameter), was observed during the proliferative phase (Figure 10D); in some sections of the urogenital sinus, up to 80 cell layers were observed. Slight luminal dilatation was also described. The stratum lucidum consisted of a layer of flattened translucent cornified cells (12–15 µm in diameter, not possessing a nucleus).

Only one pair of vestibular glands were collected during the proliferative phase of the koala estrous cycle. The epithelium of the vestibular glands in the proliferative phase of the ovarian cycle of the koala was composed of hyperplastic columnar cells interspersed with the occasional goblet cell. The lumina of these glands were filled with degenerating epithelial cells, secretory material, cellular debris, and leukocytes. Basophilic secretory vesicles were also observed in the apical cytoplasm of some epithelial cells.

### 3.5. Ovarian and Reproductive Tract Histology of the Koala Luteal Phase

**Figure 11:**
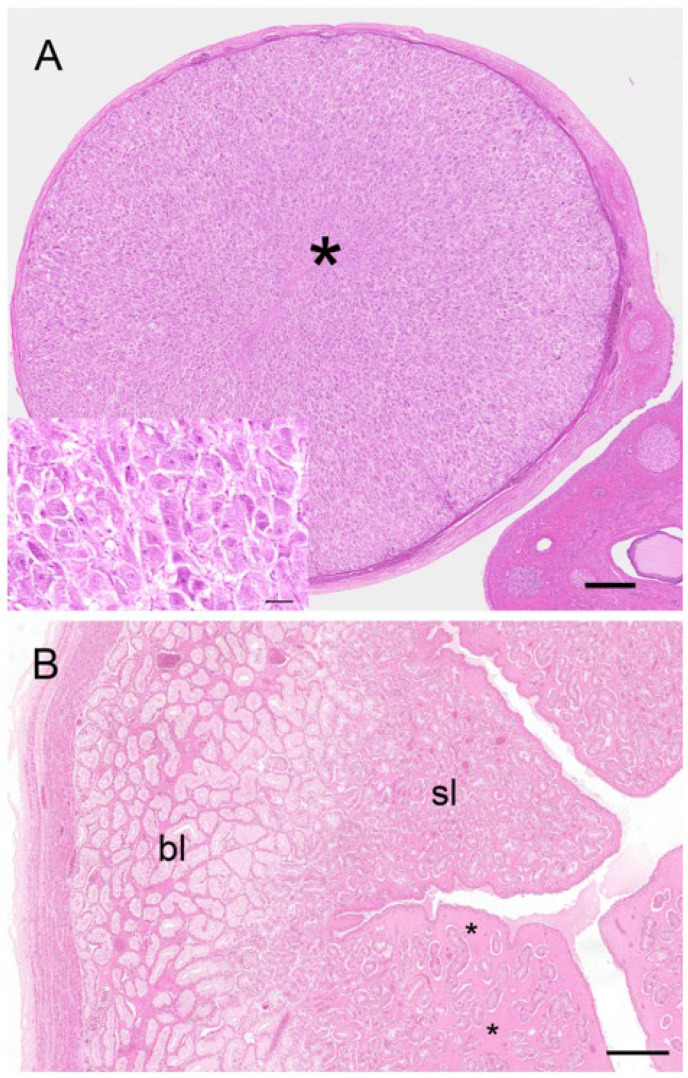
Histological features of ovary (**A**) and uterus (**B**) from pregnant koalas. (**A**) Large corpus luteum (asterisk), consisting of numerous polygonal cells with abundant eosinophilic granular cytoplasm (inset). HE. Bar = 1 mm (inset—Bar = 50 µm). (**B**) Bi-layered appearance of the uterus, with a basal layer consisting of glands lined by epithelial cells with a pale eosinophilic cytoplasm (bl), and a superficial layer (sl) with densely packed glands lined by epithelial cells with a hypereosinophilic cytoplasm. Note the interstitial edema (asterisk) in the superficial layer. HE. Bar = 600 µm.

Two reproductive tracts were recovered from pregnant koalas estimated to be at the pharyngeal arch stage and considered to be in the last third of gestation. The corpora lutea associated of these pregnant tracts measured 7.3 mm and 6.5 mm in diameter, respectively. Histologically, each CL consisted of a theca externa which measured approximately 45 µm thick and which encapsulated a solid mass of luteal cells (Figure 11A). There were also prominent blood vessels, grossly observable on the surface of the CL, which, after histological examination, were found embedded in the tissue of the theca interna. The lutein elements were presumably granulosa in origin and consisted of large polygonal cells with granulated cytoplasm, indicative of cells with secretory activity (Figure 11A, inset). Based on the developmental stage of the fetus and endocrinological data from pregnant koala progesterone profiles [15], it is likely that this CL was producing peak levels of progesterone. The respective measurements of the longest and widest dimensions of the luteal cells were 61.5 ± 3.9 mm and 37.0 ± 2.9 mm. The luteal cell nuclei were conspicuous and had a diameter of 12.0 ± 0.5 mm. Interspersed amongst the lutein cells were strands of connective tissue cells and small blood vessels. The CLs of pregnancy were both filled completely with luteal tissue.

Corpora lutea (up to 8 mm in diameter) were also observed from non-pregnant koalas as large spherical structures, grossly protruding from the surface of the ovary and surrounded by connective tissue. The “large luteal cells” of the corpus luteum of non-pregnancy were polyhedral, with an approximate diameter of 30–50 µm, and possessed a large spheroidal nucleus (7–9 µm in diameter) and multiple lipid granular inclusions in the foamy eosinophilic cytoplasm. The stroma of the corpus luteum was formed by a few collagen fibers and many reticular fibers located around the structure together with small blood vessels. The “small luteal cells” had an approximate average diameter of 15 µm and possessed an irregular-shaped nucleus (5–8 µm in diameter) and multiple lipid droplets in the cytoplasm.

Although the glandular ipsilateral gravid uterus of the two pregnant koalas observed in this study was grossly larger than the contralateral non-gravid uterus, the endometrial (4.3 mm) and myometrial (0.8 mm) layers of the gravid uterus were smaller than those of the non-gravid uterus (6.3 mm and 1.2 mm), respectively. However, the most notable aspect of the endometrial tissue in both uteri was its bi-layered appearance (Figure 11B). The basal layer of the endometrium was composed of non-ciliated, hyperplastic, glandular epithelial cells; these cells contained basally located nuclei and eosinophilic cytoplasm. The lumina of the glands of the basal endometrium contained secretory products, referred to by Shorey and Hughes [17] as uterine milk. The submucosa, in the region of the basal endometrium, was filled with a homogeneous eosinophilic material and blood vessels. A superficial layer of the endometrium was clearly differentiated from the basal layer by an increase in density of the glandular tissue and a marked increase in cytoplasmic eosinophilia. The nuclei of these cells were centrally located, and the lumina of glands in this region appeared narrower. Although both gravid and non-gravid uteri of the pregnant koala exhibited a bi-layered appearance, the basal layer of the gravid uterus was not as pronounced (1.0 mm) as that of the non-gravid uterus (2.6 mm). The most distinctive histological difference between the two uteri was the increase in the degree of oedema associated with the superficial layer of the non-gravid uterus (Figure 11B).

The observation of non-pregnant koalas revealed that both glandular uteri of the non-pregnant luteal phase were similar in size and histology. The myometrium consisted of an outer longitudinal layer and inner circular layer which measured 0.1 ± 0.03 mm and 0.3 ± 0.01 mm thick, respectively. The endometrium was 3.8 ± 0.6 mm thick and was composed of basal and superficial layers of 1.9 ± 0.2 mm and 1.9 ± 0.1 mm, respectively. The histology of the endometrium was similar to that described for the pregnant uteri, although there was not the same degree of oedema or presence of eosinophilic secretory material in either the superficial or basal layers. The glandular epithelial cells of the basal layer measured 57.0 ± 2.5 µm high, while those of the superficial layer were 44.3 ± 1.5 µm high. The uterine endometrial epithelium also showed frequent vacuolar degeneration and high mitotic activity, with associated detachment of the most external layer and presence of cellular debris and leukocytes (neutrophils) in the uterine lumen.

The histological features of the vaginal cul-de-sac of non-pregnant koalas during the luteal phase were similar to the ones of the interestrous stage, lined with a single layer of epithelial cuboidal or columnar cells. The muscularis of the lateral vaginae was thickened during the luteal phase to such an extent that the lumen of the canal was constricted. The epithelium lining the lateral vaginae was composed of five to nine layers of non-cornified cells and showed some evidence of deterioration. There was an accompanying variable polymorphonuclear cells infiltration. The epithelium lining the urogenital sinus in the non-pregnant luteal phase showed hyperplasia of the layers. Cornified epithelium was still observed in some specimens that also showed diffuse edema of the submucosal layers. The submucosa and muscularis of the urogenital sinus were heavily vascularized. The epithelial lining of the urogenital sinus of the koalas in this phase was composed of three to five layers of non-keratinized cuboidal cells.

**Figure 12:**
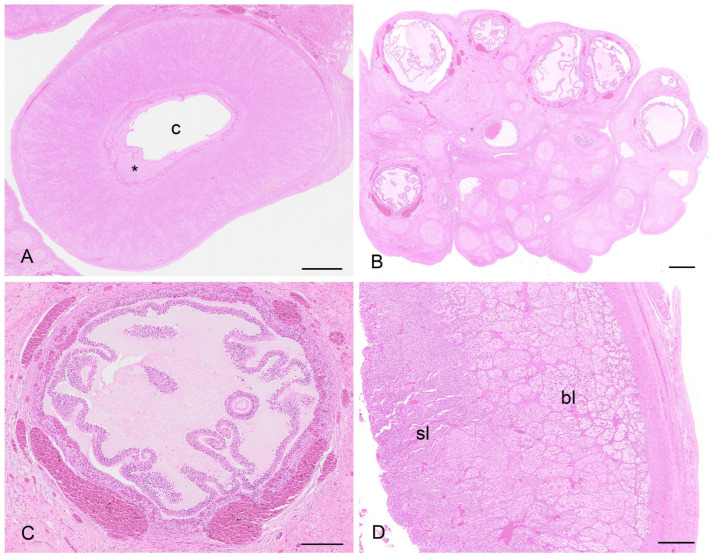
Histological features of the ovary (**A**–**C**) and uterus (**D**) from a post-partum koala. (**A**) Corpus luteum with a central cavity (C) lined by an amorphous eosinophilic material (asterisk). HE. Bar = 1 mm. (**B**,**C**) Numerous Graafian follicles in the process of degeneration in a post-partum ovary. Higher magnification of a generated follicle in (**C**)—note the irregular detachment of the stratum granulosum. HE. Bar = 1 mm (**B**), 200 µm (**C**). (**D**) Reduced thickness and regression of the superficial (sl) and basal layer (bl). HE. Bar = 500 µm.

There were two koalas in this study which were processed for histology that showed evidence of patent birth canals that were presumed to have recently given birth, one with a pouch young and one without. The CLs from both reproductive tracts were hollow, and the cavity was lined by a layer of eosinophilic homogeneous substance (Figure 12A), beneath which was a connective tissue layer, presumably laid down by the original theca interna, which measured 250 µm to 325 µm thick. The outer edge of the mature CL was lined by what appeared to be the original theca externa and measured 125 µm to 210 µm thick. The majority of the CL was composed of eosinophilic polygonal luteal cells of slightly shrunken appearance, surrounded by a blood capillary network. The longest and widest dimensions of cells from the CL of each koala were 36.3 ± 1.8/20.8 ± 1.5 mm and 56.3 ± 3.0/31.3 ± 3.0 mm, respectively. The cell nuclei of both CLs were small and pyknotic and had mean diameters of 8.0 ± 0.5 and 10.8 ± 0.5 mm, respectively. Based on their size and the lack of other structures which resembled CLs, there was little doubt that these CLs were those of a recent pregnancy.

The contralateral ovaries of both recently post-partum koalas also showed evidence of follicular atresia. Each ovary had numerous Graafian follicles (2 to 3 mm in diameter) in the process of degeneration (Figure 12B,C). It is likely that these Graafian follicles grew during pregnancy but became atretic during the latter stages of pregnancy or very early post partum. The granulosa cells of the more mature follicles in these ovaries were in the process of collapsing away from their basement membrane. Blood vessels were prominent in the stromal tissue immediately outside the theca and within and between the cells of the theca externa and theca interna. Other follicles in the same ovaries showed evidence of slightly more advanced atresia, as indicated by the rupture of thecal blood vessels and the influx of red blood corpuscles into the antrum of the follicle. One female koala died when her pouch young had a head length of approximately 12.0 mm. We estimated a head length of 12.0 mm corresponded to a pouch young age of approximately 1 to 2 weeks. The ovary supporting the old CL of pregnancy in this animal contained numerous Graafian and blood-filled follicles. Although a solid structure, the CL consisted of luteal cells in the process of regression which were surrounded by invading connective tissue and blood vessels.

At the time of death, a captive koala had a pouch young estimated to be approximately 4 to 6 weeks old. While the ovary of this animal had numerous Graafian and hemorrhagic follicles, the luteal cells of the old CL were almost completely regressed. Connective tissue had thoroughly invaded the CL, forming a lattice network around what were originally luteal elements, but which had now become hollow cavities. The CL of a second captive female which died 57 d after giving birth had regressed to 2.3 mm in diameter. Numerous Graafian follicles had formed on both ovaries, some of which had become atretic and formed blood-filled follicles. It is likely that this female had passed through at least one interestrous period since the pregnancy and prior to her death.

The uteri of both recently post-partum koalas showed evidence of a marked regression of both superficial and basal endometrial layers (Figure 12D). The nuclei of cells in both layers were pyknotic, and the cytoplasm vacuolated and degenerated. The uterine lumen was devoid of epithelium. In the uterus of one koala, retained fetal membranes were still present. Approximately 12 d post-partum, both glandular uteri in one koala had regressed such that they were not histologically different from interestrous uteri.

The vaginal cul-de-sacs of both recently post-partum koalas were similar to those in the interestrous phase. The lateral vaginae, however, appeared thickened, similar to those observed during the luteal phase. The urogenital sinuses of the same koalas were lined with five to nine layers of non-cornified epithelium. There was also evidence in both animals of recent trauma to the mucosa of the urogenital sinus, the submucosa of which was heavily vascularized.

**Figure 13:**
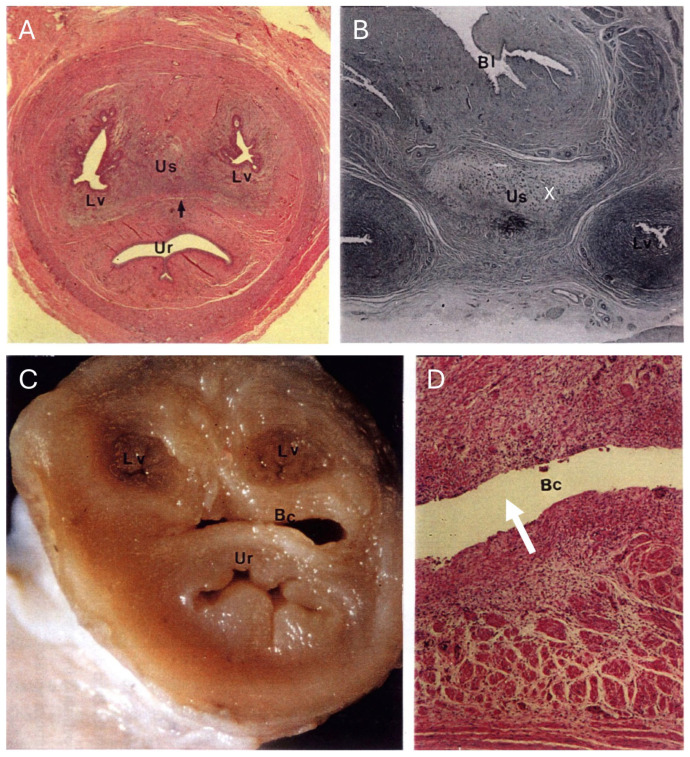
(**A**). Transverse section through the koala urogenital strand during interoestrus (11.5×); (**B**). Transverse section of the central portion of the urogenital stand tissue of a pregnant koala showing oedema associated with the formation of the birth canal (11.5×); (**C**). Slit-like birth canal found in the urogenital strand of a recently post-partum koala (11.5×); (**D**). The birth canal of the koala showing no evidence of an epithelial lining (76×). Bc—Birth canal; Bl—Bladder; Lv—Lateral vagina; Ur—Urethra; Us—Urogenital strand; Black arrow—possible weak point in the connective tissue of the urogenital strand where the birth canal first forms; White “X”—Evidence of oedema formation; White arrow—Transverse tear in urogenital strand.

In both recently post-partum koalas, a presumptive patent birth canal was still present. The birth canal was formed in the connective tissue as a slit-like lumen between the lateral vaginae and urethra (Figure 13A–D). In a transverse section of the urogenital sinus in one koala, the birth canal measured 4.8 to 4.9 mm wide and 0.7 to 0.85 mm deep. There was no epithelium lining the lumen of the canal. In the female which had presumably given birth most recently, the lumen of the canal was large and defined by the distribution of adjacent muscle, lateral vaginae, and the urethra. A second female showed evidence of partial tissue repair in the region of the birth canal, with the lumen of the canal appearing to be in the process of closure. The tissue filling the birth canal was composed of loose connective tissue, muscular bundles, blood vessels, leukocytes, and extravasated red blood cells. There was no evidence of retained fetal membrane remnants. A birth canal was not present in the urogenital strand of a female koala 1 to 2 weeks post partum, suggestive of the rapid closure of the canal post partum. The urogenital strand tissue in this animal was in a state of re-organization and was composed of loosely arranged connective tissue, blood vessels, and small bundles of muscle fibers.

## 4. Discussion

The microanatomy of the koala ovary is fundamentally similar to that described for other marsupials [18,19,20,21,22,23] and eutherian mammals [24,25,26,27]. A central medulla containing blood vessels, nerves, and connective tissue is surrounded by a cortex in which various stages of germ cell development (primordial, primary, secondary, and tertiary follicles), interstitial tissue, and depending on the stage of the cycle, a functional or degenerating corpus luteum are found. Given that ovulation in the koala is induced by coitus [28], it is possible to recognize three potential pathways for the fate of the mature pre-ovulatory follicle and oocyte. These pathways are dependent upon the success or otherwise of the mating stimulus and include (1) growth and atresia of the pre-ovulatory follicle associated with an anovulatory cycle, (2) induced ovulation, formation of a CL, and presence of a conceptus (pregnancy), and (3) induced ovulation, formation of a CL, and absence of a conceptus (non-pregnant luteal phase).

### 4.1. Anovulatory Cycle

It was not possible to ascribe ovarian or reproductive tract histology representative of the anovulatory phase of the koala in this study. However, according to Brambell [24], a lack of coital stimulus in the estrus rabbit, for which ovulation is also induced, leads to an “over-ripening” of mature follicles and subsequent atresia resulting in the formation of blood-filled follicles. The koala ovary in all stages of the reproductive cycle showed evidence of atretic and blood-filled or hemorrhagic follicles; the phenomenon of follicular atresia will be discussed below.

### 4.2. Induced Ovulation, Formation of a CL, and Presence of a Conceptus (Pregnancy)

The second potential fate of a koala oocyte is that coitus results in ovulation, fertilization is successful, a CL is formed, and pregnancy develops. O’Donoghue [29] previously described in detail the formation of the CL from 10 pregnant females. In the present study, it was not possible to conclusively confirm O’Donoghue’s [29] earlier observations that granulosa cells of the ovulated follicle give rise to the bulk of the luteal tissue. However, in some histological sections of CL, it was possible to observe what appeared to be the cells of the theca interna migrating through luteinized granulosa tissue to line the inner edge of the antrum. Interestingly, while O’Donoghue [29] found that the CL of pregnancy was hollow in all 10 pregnant tracts he examined, the two CLs of pregnancies described in the present study were both solid structures. There appears, therefore, to be some considerable variability in the ultimate morphology of this structure. Whether or not a central cavity in the CL is present may have more to do with the final size of the pre-ovulatory antrum. Given that the koala has a mean length of estrus of approximately 10 d [16], it is feasible that females which mate early in their estrus have smaller pre-ovulatory antra than those which mate later in their estrus. A solid CL may, therefore, be a result of luteinized granulosa cells occupying the antral space left by a smaller pre-ovulatory follicle. Conversely, the hollow CL found in some recently post-partum females in this study may simply be related to larger sized pre-ovulatory follicles, such that hypertrophy and hyperplasia of the granulosa and thecal cells are simply not rapid enough to fill the antrum of the pre-ovulatory follicle during the luteinization period. Nevertheless, there appears to be little doubt that the CLs described by O’Donoghue [29] were not luteinized un-ovulated follicles, as they were the only notable structures on either ovary and all females were carrying a fetus.

The luteal cells forming the CL of pregnant koalas were larger than those of non-pregnant or recently post-partum koalas, and showed cytological evidence of increased secretory activity [2]. The fetuses of both pregnant koalas observed in this study were determined to be in the last third of their embryonic development. The profiles of peripheral progesterone through pregnancy reported in Johnston et al. [16] have shown that progesterone production is at peak production during this period. The CLs of recently post-partum koalas contained luteal cells that were smaller in size than those of the CL of pregnancy. The nuclei of these cells were pyknotic, and their cytoplasm showed little evidence of secretory activity. By 2 weeks post partum, the blood vessels supporting the CL had broken down, and strands of connective tissue had started to invade the spaces left by shrinking luteal cells. After 30 to 40 d, the CL had been further invaded with connective tissue, and the majority of luteal cells had degenerated. Fifty-seven days post partum, the presumptive CL of pregnancy had regressed substantially to about one quarter the diameter of the CL of pregnancy.

### 4.3. Induced Ovulation, Formation of a CL, and Absence of a Conceptus (Non-Pregnant Luteal Phase)

The third possible fate of the koala oocyte is that ovulation is induced successfully, but fertilization and/or pregnancy is not successful. While the progesterone profiles during the luteal phase of mated but non-parturient koalas are similar to those of pregnant cycles [16], the CL of some non-pregnant koalas in the current study were larger than those of pregnancy, and all showed secretory activity. Without exception, all female koala reproductive tracts representative of the non-pregnant luteal phase showed evidence of uterine hyperplasia, but whether such changes in uterine morphology constitute a state of physiology equivalent to pregnancy (maternal recognition or not) requires further investigation.

### 4.4. Folliculogenesis

In this study, koala ovaries associated within the proliferative phase had Graafian follicles ranging from 4 mm up to approximately 7 mm in diameter. While oocytes were aspirated from two 6 mm follicles, no polar bodies or metaphase spindles were detected. Failure to observe these structures may be related to autolytic post-mortem changes. The presence of Graafian follicles 4 mm or greater in the koala ovary were clearly associated with the proliferation of the reproductive tract, presumably a consequence of estrogen secretion. These large, probably proestrus koala follicles appear to be amongst some of the largest recorded of any marsupial, and an outlier to the theory proposed by Perry [29] and Tyndale-Biscoe and Renfree [30] that pre-ovulatory follicle size is positively correlated with adult body size. In a laparoscopic study of pre-ovulatory follicular development and ovulation in the Brushtail Possum (*Trichosurus vulpecula*), Crawford et al. [31] also recorded pre-ovulatory follicles up to 6 mm in diameter. A 5–7 mm diameter follicle should be detectable using trans-pouch ultrasonography, and therefore potentially useful for the purposes of predicting and diagnosing ovulation and directing artificial insemination into the ipsilateral uterus. It is also important that koala preovulatory follicles are not confused with the ultrasonography of early-stage ovarian bursitis.

### 4.5. Zona Pellucida

The zona pellucida of the koala oocyte was unusual, being over twice as thick (14 to 15 µm) as reported for other marsupials (1 to 6.3 µm) [30] but similar to that of eutherian mammals [32]; this also includes the wombat, the koala’s close phylogenetic relative (10–13 µm; [33]). Rodger and Mate [34] commented that the zona pellucida of opossums and possums is diffuse and broad before ovulation, but becomes condensed and thinner following ovulation. Whether the zona pellucida of the koala retains its extreme thickness at the point of fertilization is still to be determined. The thickness of the zona pellucida in the koala raises some interesting questions as to the mechanism of penetration by the spermatozoon. Given that the koala acrosome is located within a groove along the inner curvature of the hook-shaped head [35], it is interesting to speculate how the sperm head (11.8 µm in length; [36]) might effectively penetrate the zona pellucida during acrosome reaction and fertilization.

### 4.6. Follicular Atresia

Follicular atresia is the process whereby ovarian follicles undergo degenerative changes, resulting in the loss of the oocyte and follicular integrity. While follicular atresia can occur at all stages of the reproductive cycle, the sometimes poor fixation of koala ovarian tissue in this study may have obscured subtle degenerative morphological changes typical of atretic follicles in the very early stages of folliculogenesis; consequently, further studies with better fixed tissue are required to document koala ovarian dynamics in more detail. Nevertheless, a large number of hemorrhagic follicles were observed in the koala ovaries examined in this study, particularly those ovaries in the interestrous phase. Similar structures have also been reported in rabbit ovaries [24,37], and attributed to atresia resulting from an over-ripening of the pre-ovulatory follicle. While “over-ripening” of a preovulatory follicle in association with an anovulatory cycle was not directly identified during the interestrous phase of the present study, a similar atretic process to that described in the rabbit was observed in the ovaries of two koalas which had recently given birth (post-luteal phase). Both koalas had large regressing CLs on one ovary but mature atretic follicles on the other, that showed evidence of hemorrhagic infiltration and granulosa collapse. It is likely that these follicles may have formed during pregnancy, but had been prevented from further development by the high levels of progesterone associated with the pregnancy or by the inhibiting suckling stimulus of the pouch young [15]. It is proposed, but requires further validation, that the atretic cellular dynamics described in the ovaries of recently post-partum koalas are similar to those which cause atresia of mature follicles in non-mated estrus cycles. What was observed histologically as hemorrhagic follicles without a granulosa layer, we suggest, are likely to be follicles in which the granulosa layer has collapsed into the antrum of the follicle from blood released from the breakdown of capillaries that previously supported folliculogenesis.

In this study, the membrana granulosa and theca interna of atretic follicles also showed varying degrees of hyperplasia or what is commonly known as luteinization. The luteinization of atretic theca interna has been described in a variety of species including the rabbit [37], water shrew (*Neomys fodiens bicolor*) [38], and rat [29]. Interestingly, Harrison Matthews [39] and Woolley [40] have also described the luteinization of unovulated follicles in the ovaries of the Matshie’s tree-kangaroo (*Dendrolagus matshiei*) and the brown Antechinus (*Antechinus stuartii),* respectively. While atresia and hemorrhaging of large Graafian follicles in the koala can be histologically spectacular, it should be remembered that the atretic process can also occur with immature follicles, some which may possess only a small antrum or no antrum at all. Although not documented in the figures, numerous examples were observed of atretic immature follicles showing hyperplasia of the theca interna.

### 4.7. Origin of the Interstitial-like Tissue in the Koala Ovary

In 1916, O’Donoghue recognized that thecal cells found in atretic koala follicles may take on a luteinized appearance. However, he was adamant that the theca was not the source of ovarian interstitial tissue. In the present study, there was evidence to indicate that the theca interna, and less commonly the membrana granulosa of unovulated follicles, underwent hyperplasia and were ultimately transformed in what might be described as ovarian “interstitial-like” tissue. A similar process of interstitial tissue formation has also been reported for the Virginia opossum (*Didelphis virginiana*) [41].

It is unlikely that the “interstitial-like” tissue in the koala ovary was formed from old CLs, as luteinized thecal cells were clearly distinctive, being oval shaped, less eosinophilic, and smaller than the luteal cells of CLs. Two types of “interstitial-like” tissue cells were described in the present study. These cell types may represent cells of the theca interna and membrana granulosa, respectively. Alternatively, it is possible that both cell types are thecal in origin, but that the larger cells are further advanced in their hyperplastic transformation.

Preliminary observations in this study, therefore, suggest that “interstitial-like” tissue in the koala ovary is not laid down early in embryonic development, but rather results from the luteinization of atretic follicles. No interstitial tissue was found in a 6-month-old pouch young, but the theca interna of atretic follicles in this same ovary showed evidence of hyperplasia. There was also little evidence of interstitial tissue degeneration or breakdown in any of the koala ovaries examined, suggesting that this tissue may become permanently incorporated into the ovarian stroma. This may also explain the high proportion of encapsulated “interstitial-like“ tissue found in the ovaries of some koalas.

A study on the structural features of the southern hairy-nosed wombat (*Lasiorhinus latifrons*) [42] revealed that the ovary of this species also contained distinctive lobules of luteinized interstitial cells, many of which contained PAS-positive material similar in appearance to the interstitial tissue in the koala ovary. Breed et al. [42] have also suggested that the PAS-positive material described in the cortex of the wombat ovary may have been partly formed from the zona pellucida that surrounded the oocytes in developing follicles, noting the presence of zona pellucida remains in many of these luteinized lobules, but later in the same paragraph, they go on to state that the luteinized tissue has arisen from atretic follicles. In the current study, there was abundant evidence that the lobules of the koala interstitial tissue were associated with luteinization of the granulosa and theca internal cells of what appeared to be atretic follicles, with no evidence of zona pellucida involvement.

Ovulation in the koala is induced by a combination of coital stimulation and seminal biochemistry [28], whereas ovulation in the wombat appears to be spontaneous [43]. While we had originally considered that the source of the interstitial tissue in the koala may have arisen from atretic follicles formed following a failure of ovulation, the similar presence of this tissue in both the wombat and koala suggests otherwise. Rather, these lobules of interstitial cells, commonly found in the ovaries of both species, may in fact be a coincidental consequence of the luteinization of immature follicles following the LH surge of ovulation. Clearly, the ovarian luteal dynamics of both species require much further investigation.

### 4.8. The Function of Interstitial Tissue in the Koala Ovary

So, what of the function of the “interstitial-like” tissue in the koala ovary, or does it have a function? Perhaps it has a direct or indirect role in estrogen production. In describing the ovary of the pocket gopher (*Geomys bursarius*), Mossman [44] showed that thecal gland hypertrophy coincided with the period of maximal ovarian estrogen output. Stafford et al. [45] later suggested that this tissue in the guinea pig was the principal source of estrogen [45]. Rat interstitial tissue formed from theca interna of atretic follicles has also been shown to be capable of producing estrogen and androgen in response to hormonal stimulation [29]. It is well accepted that under LH stimulation, the thecal cells of the growing follicle can produce androgens which can be further processed by granulosa cells to produce estradiol-17β [46]. Perhaps the “interstitial-like” tissue in the koala ovary also acts as an indirect source of estradiol-17β, supporting the growing pre-ovulatory follicle.

The rabbit ovary contains large amounts of interstitial tissue [47]. According to Hilliard et al. [48], the cells of this tissue contain large stores of cholesterol, which become mobilized by LH to secrete 20α-dihydroprogesterone following coitus but prior to ovulation. Hilliard et al. [49] have suggested that 20α-dihydroprogesterone acts as a positive feedback agent in the rabbit to prolong and heighten LH discharge after mating. While this particular functional role of 20α-dihydroprogesterone has been questioned by Goodman and Neill [50], there are, nevertheless, similarities in the reproductive [12] and ovarian cellular dynamics of the koala and rabbit (for example, ovulation in the koala and rabbit is induced by coitus). It would be interesting, therefore, to determine whether 20 α-dihydroprogesterone or any progestogen metabolite can be located in koala interstitial tissue. Handasyde et al. [51] have previously identified high concentrations of 20-hydroxyprogesterone in the peripheral circulation of the koala, but ascribed no functional role to this hormone.

Given the hyperplastic appearance of the atretic theca interna described in this study, it is difficult to understand how a luteinization-like process could occur without a surge of LH, especially given that ovulation in the koala appears to be induced by coitus [28]. Johnston et al. [16] have shown that female koalas which do not mate exhibit shortened (presumably anovulatory) cycles, and that estradiol concentrations during these cycles rise, coincident with behavioral estrus. It is possible that rising estradiol levels produced in the pre-ovulatory follicle result in the release of LH via a feedback loop to the hypothalamus, but that the concentration of LH produced in this “passive” way is not enough to induce ovulation. In other words, while a sub-threshold level of LH might be enough to induce the luteinization of cells of the theca interna of atretic follicles, stimulation of the “copulo-ceptive reflex arc” [28,52] may be required in order to cause a LH surge large enough to trigger ovulation.

### 4.9. Oviduct

Previous descriptions of the koala reproductive tract [3,5,10] have not recognized the four standard regions of the mammalian oviduct [53], referring only to plicated and non-plicated portions. In this study, all four regions of the oviduct were distinguished, including a fimbriated infundibulum, a thin-walled ampulla, a convoluted isthmus, and extramural/intramural utero-tubal junction. However, to facilitate comparison with MacKenzie [3] and Brown [5], the measurements of the length of the non-plicated (ampulla) and plicated (isthmus) regions of the oviduct in this study were based on gross morphology, rather than on the structure of the luminal epithelium. The non-plicated and plicated oviduct lengths reported in this study were, respectively, shorter and longer than those recorded by MacKenzie [3] and Brown [5]. Nevertheless, the predominantly cuboidal epithelium of the fimbriated folds of the infundibulum contained considerable numbers of both ciliated and goblet mucous-producing cells. The ampulla of the koala oviduct was clearly defined by its thin walls and a marked proliferative branching of the many smooth longitudinal folds of the mucosa. Such a complex labyrinth is typical of ampullae found in most species of mammals [53], where it is usually the site of fertilization and early embryonic development. The isthmus region of the koala oviduct was characterized by an increase in the thickness of the muscularis, a reduction in the number of longitudinal folds of the mucosa, and a corresponding decrease in the degree of corrugation of the longitudinal folds. While it may be a post-mortem artefact, a few ciliated or goblet cells were found in the essentially cuboidal epithelium of the ampulla or isthmus of koalas in the interestrous phase of the reproductive cycle. However, ciliated cells were observed lining the lumen of the ampulla and isthmus of koalas during estrus. While it was difficult to distinguish the extramural junctura from that of the terminal portion of isthmus, both macroscopically and histologically, the intramural juncturae were easily identified as their lumen was markedly constricted compared to that of the lumen of the uterus. Sections through the region of the utero-tubal junction failed to identify with clarity any protective uterine folds forming a utero-tubal barrier. The histology of the epithelium lining the lumen of the ampulla and isthmus regions of the oviduct from koalas in estrus showed significant hyperplasia. Similar hyperplasia of the koala oviduct has also been observed by Handasyde et al. [11] after injection of estradiol. It is highly likely, therefore, that changes in the cytology of the oviduct approaching and during estrus are mediated by an increase in estradiol concentration produced by the pre-ovulatory follicle.

### 4.10. Uteri and Cervix

The shape of the paired uteri of the koala was typical of other monovular marsupials, being fusiform and joined caudally by a sheet of peritoneum. As Mackenzie [3] originally noted, the uteri of the koala have two distinct portions, an upper glandular section and a muscular cervix. The general shape of the glandular portion of the uteri changes dramatically with the stage of the reproductive cycle, being larger in volume during the luteal phase, but greatest during pregnancy (Figure 2). The muscular cervix in the koala was relatively long (15 mm) and is likely to represent a significant obstacle for the neonate (16 to 17 mm in length) to traverse during parturition and its ultimate passage to the pouch.

The histology of the myometrium, endometrium, and epithelium of the upper glandular and cervical portions of the uterus in the interestrous koala was unremarkable and similar to that reported for other marsupial species [31]. The uterine cervices protrude substantially into the upper vaginal cul-de-sac, and it is possible that crypts formed from the increased folding of the epithelium in this region at estrus may serve as potential receptacles for sperm storage. Degenerating spermatozoa were recovered from this region of cul-de-sac in one estrous female [12]; the reproductive tract of this female was in a proliferative condition, and one ovary contained an unovulated follicle of 5 mm diameter.

The changes in the volume and histology of koala uteri during the reproductive cycle were similar to those of most marsupials [31]. During late pro-estrus and estrus, there was a slight but significant symmetrical increase in the volume of both uteri, which was co-incident with an increase in the volume and development of the glandular endometrium. During estrus, the glandular lumina were patent, and the epithelium contained ciliated cells. The development and initial hyperplasia of the glandular epithelium is, therefore, likely to be estradiol-sensitive, but as in *T. vulpecula* [17], probably requires progesterone to attain maximum development.

One pregnant uterus examined was 14 times the mean volume of interestrous uteri. Although the gravid uterus was larger than the contralateral uterus, the increase in size was primarily due to the increased volume associated with the fetus and its fetal membranes and fluid. Luteal and early post-luteal uteri were also up to two to three times larger in volume than interestrous uteri. The general increase in size of luteal uteri was principally due to hyperplasia of the glandular epithelium and increased edema of the endometrium, particularly of the basal region, presumably associated with progesterone secretion [16]. The superficial and basal regions of uterine glands of the koala noted in this study have also been described in the pregnant uteri of *T. vulpecula* [17]. These authors were uncertain about the functional role of the superficial layer, suggesting it may be responsible for the production of shell membrane. Shorey and Hughes [17] believed that the basal glandular epithelium was the functional tissue concerned with the active secretion of uterine milk for the maintenance and expansion of the unattached embryonic vesicle. They also reported that during the late luteal and post-luteal phases, the cytoplasm of cells of the basal glandular epithelium showed evidence of rupture and later appeared necrotic. Similar histology has been reported in this study in the uteri of recently post-partum koalas. This phenomenon is likely to be the result of earlier progesterone hyper-stimulation, and the subsequent apocrine and holocrine secretion of large amounts of the glandular component of ‘uterine milk’. The histology of the post-luteal glandular uterine epithelium in koalas was similar to that described for *T. vulpecula*, in that the uteri involute and the endometrium degenerates to a condition comparable with interestrous. However, in this study, there was no light microscopic evidence of uterine gland regeneration as reported for *T. vulpecula* [54].

### 4.11. The Vaginal Complex

Tyndale-Biscoe and Renfree [31] have commented that the vaginal complex region of the marsupial is by far the most variable component of the female’s reproductive anatomy. However, compared with other marsupials, the vaginal complex of the interestrous koala was unremarkable. Although Brown [5] described the cranial opening of the lateral vagina into the vaginal cul-de-sac as occurring 5 mm caudal to the tip of the cervix, similar measurements recorded in this study showed that the relative positions of the cervical and lateral vaginal ostia were highly variable. The vaginal cul-de-sac was divided permanently by a thick muscular septum, and none of the koalas examined in this study showed evidence of incomplete medial septa of the cul-de-sac, as has previously been reported by Lee and Martin [6] for Victorian koalas. During the proliferate phase of the reproductive cycle, the vaginal cul-de-sac showed marked gross and histological changes. The volume of the cul-de-sac also increased, and the previously cuboidal epithelial cells of the interestrous cul-de-sac were transformed into tall hyperplastic columnar cells. Histological evidence from the present and previous studies [5,10] suggests that the cytology of lateral vaginae and vaginal cul-de-sac epithelia is different. Such a demarcation appears consistent with the original reproductive tract definitions of Forbes [1] and Mackenzie [3], and likely reflects a difference in the functional anatomy of these two reproductive tract components. It should be noted that the complete separation of the left and right sides of the reproductive tract, afforded by the medial septum, also implies that the successful delivery of spermatozoa (naturally or via artificial insemination) must occur into the ipsilateral horn to the ovary of ovulation.

### 4.12. Lateral Vaginae

In contrast to the findings of MacKenzie [3] and Brown [5], who suggested that the lateral vaginae begin caudally before the urethra, observations from this study revealed that both structures form at a similar distance along the urogenital sinus. The lateral vaginae of interestrous koalas are relatively short in length and straight when compared to other marsupials [5,31]. While the histology of the lateral vagina in the interestrous phase was also similar to that described for other marsupials [31], there was hyperplasia of the squamous epithelial cells lining the vaginal lumen and an increase in the thickness of the muscularis during the proliferate phase. During pregnancy, luteal, and early post-luteal phases, the cross-sectional diameters of the lateral vaginae were increased, but the lumina of these structures were constricted; a similar finding has been reported for *T. vulpecula* [55]. While MacKenzie [3] and Brown [5] reported that the urogenital sinus of the koala was slightly wider caudally, no mention was made of a cranial expansion of the urogenital sinus, the “urogenital swelling”. Most noticeable in the reproductive tract of the interestrous koala, the “urogenital swelling” also provides a useful landmark for the relative position of the formation of lateral vaginae and urethral lumina, as well as the lower extremity of the urogenital strand. It is also possible that the thickening of connective tissue in this region is associated with the transient and repeated formation of the presumptive “birth canal” (see below, Section 4.15).

### 4.13. Urogenital Sinus

The histology of the urogenital sinus from interestrous koalas was similar to that described for other marsupials [30]. However, the histology of the urogenital epithelium during estrus showed remarkable hyperplasia and hypertrophy of the epithelium resulting in cell aggregations up to 80 layers thick. Rarely were these epithelial cells completely cornified, even during estrus. The lack of cornified epithelial cells in the urogenital sinus may explain a similar reduction in these cell types in smears obtained from the urogenital sinus used to monitor estrous cycle activity in the koala [16]. In terms of the physical dimensions of the urogenital sinus, it was interesting to note that the erect penis (85 mm; [12]) of the koala was over twice the length of the urogenital sinus plus the common vestibule (40 mm), and that this disparity in size, along with the vigorous thrusting of the keratinized penile spines, may contribute to some of the urogenital trauma associated with mating (Johnston, *personal observations*). During or following mating, it is not uncommon to observe blood or a blood-stained discharge dripping from the female’s common vestibule. Marked proliferation of the urogenital sinus epithelium at estrus may provide important protection for the mucosa against the spines of the glans penis during mating. It is possible that females which do not exhibit significant proliferation of their epithelium during estrus are more susceptible to mucosal trauma during coitus. It is also likely that the stretching of the urogenital sinus by the thrusting of the male’s penis during copulation is important for stimulating the ‘copulo-ceptive reflex arc’ responsible for the subsequent hypothalamic release of LH from the adenohypophysis to induce ovulation [28] or to possibly increase mucosal surface exposure to ovulating factors in the koala semen.

### 4.14. Vestibular Glands

Exocrine glands associated with the lower urogenital sinus in the female koala have not been previously reported. Although paracloacal glands have been reported in a variety of marsupials [56,57,58], descriptions of vestibular or Bartholin’s glands appear to have been largely overlooked. The glands described in this study appear to open directly into the urogenital sinus, and therefore, by definition, are not paracloacal glands, but rather vestibular glands [59]. Studies of vestibular glands in marsupials appear limited to those of Rubin [60,61], who investigated the role of sex hormones on vestibular gland morphogenesis in the American opossum *D. virginiana*. The role of the vestibular glands in domestic animals, such as the cow, is to produce mucus to lubricate the vestibule at coitus and during parturition [62]. Presumably, vestibular glands also have a similar functional role in marsupials, although this has yet to be confirmed. Brown [5] reported the presence of a white serous to mucous discharge from the urogenital sinus around the time of pro-estrus and estrus when swabbing koalas for urogenital cytology. Perhaps the vestibular glands reported in this study contribute to the discharge described by Brown [5], although this material may also come from sloughed-off epithelial tissue of the urogenital sinus associated with estrus. Interestingly, the epithelium lining the lumen of the vestibular gland in the koala becomes hyperplastic during the proliferative phase, indicating that these structures are likely to be responsive to increasing concentrations of estradiol-17β around the time of estrus [28]. The vestibular gland is supposedly a homologue of the bulbourethral or Cowper’s gland in the male, although Rubin [61] did comment that the vestibular gland in *D. virginiana* was morphologically less complex than its corresponding male homologue. While Cowper’s gland in *D. virginiana* can be divided into three components, the vestibular gland in the female is only comparable to Cowper’s gland number 3, both in terms of its relative position and histology. Interestingly, the gross morphology of vestibular glands in two female koalas described in this present study was similar to that of bulbourethral gland II found in the male koala [63]; further investigation is required to confirm this apparent homology.

### 4.15. Birth Canal

The formation and closure of the transient birth canal in the koala was similar to that reported for *T. vulpecula* [55]. The transverse sections through the urogenital strand region of interestrous koalas revealed a dense layer of connective tissues and muscle fibers. However, during the last third of pregnancy, the tissue of the urogenital strand showed marked changes in histology. In the region of the urogenital swelling, connective tissue and muscle fiber density had considerably reduced. The softening of the cervix and vaginal canal in eutherian species has been attributed to the ovarian hormone relaxin [64], but the influence of this hormone on the urogenital strand tissue of marsupials is less clear [30]. The urogenital strand connective tissue of a koala in the last third of its pregnancy showed evidence of small vacuities in the region of the prospective birth canal. It is likely that these vacuities could later coalesce to form the birth canal proper. It appears, therefore, from observations in this study, that the koala birth canal may be patent before parturition. This phenomenon is likely to assist the passage of the neonate through the urogenital strand and into the urogenital sinus. The birth canals of both koalas described in this study were not lined with epithelium. The histology of the urogenital strand region 2 to 3 weeks post partum suggests that the koala birth canal is a transient structure and is, therefore, presumably reformed at each subsequent birth. In this regard, the koala resembles the majority of other non-macropodid marsupials [31].

## 5. Conclusions

### 5.1. The Significance of This Study to the Development of Koala Artificial Insemination

There are two fundamental approaches to AI in the koala: (i) the use of a non-invasive deposition of semen as far up the reproductive tract as possible via an insemination catheter, or (ii) the introduction of semen directly into the lumen of the vaginal cul-de-sac or uterus via laparoscopy [65]. Given the relative positions of the twin lateral vaginae, non-patent birth canal, separate uteri, and the partitioning of the vaginal cul-de-sac in the koala, the deposition of semen using an insemination catheter via the cervix or even the upper lateral vaginae would represent an extremely difficult technical challenge. However, it has been possible, given the slightly dilated entrances to the lateral vaginae and the architecture of the longitudinal urogenital folds, to guide an insemination catheter into the caudal opening of each lateral vagina [65]. During estrus, the urogenital sinus shows marked proliferation of its epithelium, thereby reducing luminal diameter and increasing the presence of cellular debris. Such a change in urogenital sinus cytology makes the visualization of the lateral vaginal ostia difficult. In addition, the female needs to be anaesthetized for this procedure.

Added to the complication of negotiating the anatomy of the vaginal complex is the problem of which side of the reproductive tract to inseminate. The complete separation of both sides of the reproductive tract means that semen needs to be deposited into both lateral vaginae. As the koala pre-ovulatory follicle can reach up to 6 to 7 mm in diameter, it should be possible to detect its presence by means of ultrasonography and direct the semen into the corresponding vagina, thereby maximizing the prospects of fertilization. While the transabdominal ultrasound detection of the ovary and reproductive tract for pathology via the pouch is currently possible, the development of a suitable transrectal probe passing through the pelvic canal would further improve the visualization of ovarian structures and the documentation of ovarian dynamics. It is also possible to deliver semen in the cranial region of the urogenital sinus by means of a modified Foley catheter (Cook Australia Pty Ltd.), by which semen is forced cranially forward of the catheter balloon into both vaginae under positive displacement pressure [65].

The second approach for insemination is via laparoscopy. While this procedure requires the animal to be placed under a surgical plane of general anesthesia, it has the advantages of delivering low numbers of diluted or frozen-thawed spermatozoa directly into the upper reproductive tract. While measurements reported in this study of the distance from the external caudal rim of the pelvic canal to the midpoint of the koala uteri provide landmarks for laparoscopic insemination, this approach is also likely to be problematic and potentially dangerous, given the need to displace the large koala hindgut in order to visualize the uterus. An alternative, less risky approach for insemination could involve ultrasound-guided delivery of an insemination needle through the urogenital sinus, via the urogenital strand, into each side of the vaginal cul-de-sac.

### 5.2. The Significance of This Study to Understanding Koala Reproductive Pathology

This research emphasizes the importance of the macroscopic, microscopic, and histochemical description of the female reproductive system of koalas. The information obtained from the present study, in particular on the normal appearance and features of hemorrhagic follicles and IT in the koala ovary, made it possible to complement previous studies that have been carried out on the same species. Any knowledge that could add to a better understanding of what can be defined as “normal” and “abnormal” is crucial, especially in wild species, such as the koala, threatened by infectious diseases, such as chlamydiosis, that can cause severe pathology. The clinical diagnosis of this disease in female koalas often relies on ultrasound observation of the reproductive organs; in this instance, information regarding the normal uterine and ovarian appearance during the different stages of the estrus cycle is crucial in order to promptly detect early chlamydia-induced lesions.

## Figures and Tables

**Table 1:** Mean ± SEM lineal dimensions and comparative volumes of the component parts of the female koala reproductive tissue in the three phases of the reproductive cycle.

Koala Reproductive Tissue	Interestrous Phase	Proliferative Phase	Luteal and Post-Luteal Phase	(*p*)
Johnston [12]	N = 34	N = 8	N = 8	
Ovary				
Length (mm)	10.9 ± 0.4	12.1 ± 0.5	12.6 ± 1.2	-
Width (mm)	7.5 ± 0.3	9.3 ± 0.5	7.8 ± 0.6	-
Depth (mm)	3.6 ± 0.2	4.3 ± 0.4	4.8 ± 0.5	-
Oviduct				
Length (mm) (non-plicated)	9.1 ± 0.6	No data	7.3 ± 1.0	-
Length (mm) (plicated)	21.2 ± 1.5	No data	22.3 ± 6.8	-
Glandular uterus				
Length (mm) 1×	13.9 ± 0.4	16.4 ± 1.1	21.6 ± 0.6	-
Width (mm) 1×	6.2 ± 0.2	9.8 ± 0.5	12.6 ± 1.2	-
Depth (mm) 1×	2.6 ± 0.2	5.1 ± 0.7	7.4 ± 1.0	-
Volume (cm^3^) 2×	0.1 ± 0.2	0.4 ± 0.3	91.1 ±1.5	<0.01
Cervix				
Length (mm) 1×	13.3 ± 0.4	15.5 ± 1.3	14.1 ± 0.8	
Width (mm) 1×	4.2 ± 0.1	6.4 ± 0.5	5.8 ± 0.3	
Depth (mm) 1×	3.6 ± 0.2	6.8 ± 0.5	5.0 ± 0.4	
Volume (cm^3^) 2×	0.18 ± 0.1	0.49 ± 0.3	91.1 ± 1.5	<0.01
Vaginal cul-de-sac				
Length (max) (mm)	27.6 ± 1.1	28.5 ± 2.1	29.9 ± 2.0	
Width (max) (mm)	12.7 ± 0.6	20.9 ± 1.8	17.7 ± 1.1	
Depth (max) (mm)	3.6 ± 0.2	7.4 ± 0.5	4.9 ± 0.4	
Volume (cm^3^)	1.13 ± 0.1	3.03 ± 0.4	2.34 ± 0.3	<0.01
Lateral vaginae				
Length (av) (mm)	27.6 ± 0.7	29.5 ± 2.6	25.5 ± 1.6	
Width (av) (mm)	2.5 ± 0.1	5.1 ± 0.3	3.2 ± 0.2	
Depth (av) (mm)	2.2 ± 0.1	5.1 ± 0.3	3.1 ± 0.2	
Volume (cm^3^) 2×	0.13 ± 0.04	0.60 ± 0.6	0.20 ± 0.2	<0.01
Urogenital sinus				
Length (mm)	34.1 ± 0.9	29.7 ± 0.8	33.6 ± 2.0	
Width (mm)	6.4 ± 0.2	10.2 ± 0.5	8.0 ± 0.3	
Depth (mm)	5.3 ± 0.2	8.5 ± 0.4	6.6 ± 0.1	
Volume (cm^3^)	1.09 ± 0.1	2.42 ± 0.4	1.69 ± 0.2	<0.01
Clitoris				
Volume (cm^3^)	1.9 ± 0.1	4.1 ± 0.4	2.8 ± 0.2	0.23

av—average over entire structure, 1×—average of both left and right, 2×—volume both left and right combined.

## Data Availability

Data are available on request from S.P. and S.D.J.
